# Supplementary material for: Matching amino acids membrane preference profile to improve activity of antimicrobial peptides
Source: Commun Biol. 2022 Nov 8;5:1199. doi: 10.1038/s42003-022-04164-4 (PMC9643456; doi:10.1038/s42003-022-04164-4)
Supplement: Supplementary file 2 — Supplementary Information [file 42003_2022_4164_MOESM2_ESM.pdf]

Supporting information for:

Matching amino acid sequence to that preferred for membrane insertion improves activity of antimicrobial peptides

Shanghyeon Kim[1], Jaehoo Lee[1], Sol Lee[1], Hyein Kim[1], Ji-Yeong Sim[1], Boryeong Pak[1], Kyeongmin Kim[2], Jae Il Kim*[,1, 3]

[1]School of Life Sciences, Gwangju Institute of Science and Technology, Gwangju 61005, Republic of Korea
[2]Department of Microbiology, School of Medicine, Kyungpook National University, 680 Gukchaebosangro, Jung-gu, Daegu 41944, Korea.
[3]Pilot Plant, AnyGen, Gwangju, Technopark, 333 Cheomdangwagiro, Buk-gu, Gwangju, 61008, Republic of Korea

Table of contents

Supplement Methods 2

Supplement Figures 4

Supplement Tables 21

Supplement References 28

Supplementary Methods

Bacteria preparation, Antimicrobial peptides preparation, Minimal-inhibitory-concentration measurements, Hemolysis-activity measurements, Tryptophan fluorescence measurements, Isothermal titration calorimetry measurements, Bacteria membrane ion permeation measurements, Outer–inner membrane permeation, Field-emission scanning electron microscopy and Antimicrobial kinetics were performed as described in **Materials and Methods**.

Liposome (LUV) preparation. Bacterial-membrane-mimic DMPC/DMPG unilamellar vesicles (LUV) and mammalian-membrane-mimic PC/cholesterol vesicles with 100-nm diameters were prepared by extrusion. LiposoFast Liposome Factory Basic unit with stabilizer (Avestin, Ottawa, Canada) was used to extrude liposomes 50 times through a polycarbonate membrane filter (pore diameter, 100 nm; filter diameter, 0.75 inches) (Avestin). To prepare liposomes, 1,2-Dimyristoyl-sn-glycero-3-(Phospho-rac-(1-glycerol)) (sodium salt) (DMPG) (Avanti, Alabama, USA); 1,2-Dimyristoyl-sn-glycero-3-phosphocholine (DMPC) (Avanti); and cholesterol (Sigma, Darmstadt, Germany) were used. For circular dichroism, isothermal titration calorimetry, tryptophan fluorescence, and dynamic light scattering experiments, liposomes were reconstructed and resuspended with phosphate-buffered saline (PBS; Welgene Inc, Gyeongsangbuk-do, Korea). For calcein-permeation tests, liposomes were reconstructed with calcein solutions (80 mM, 40 mM, 30 mM, 20 mM, and 10 mM) (Sigma, Darmstadt, Germany), purified with Sephadex-G50 columns (Pharmacia biotech, Stockholm, Sweden), and resuspended with PBS.

Maximum-soluble-concentration measurements. Peptides were lyophilized and reconstructed in PBS to prepare 200 μM, 100 μM, 50 μM, 25 μM, 12.5 μM, and 6.25 μM solutions. Solubility was checked every 12 h for 5 days by comparing solution turbidity with PBS. The lowest concentration of peptide without visible turbidity was determined to be the maximum soluble concentration. Each experiment was performed at least twice.

Circular dichroism (CD) spectrum. DMPC/PG-liposome, PC/cholesterol-liposome, and peptide stock solutions were diluted in PBS. For circular dichroism, PBS with neither liposome nor peptides was also prepared. Liposomes and peptides were mixed to prepare samples (final lipid concentration, either 1000 μM or 0 μM; final peptide concentration, 20 μM). Samples were transferred into type J/1 cuvettes (Jasco, Tokyo, Japan). Cuvettes were placed into the J-815 circular-dichroism spectrometer (Jasco, Tokyo, Japan). Measurement parameters were as follows: wavelength, 190–260 nm; data pitch, 0.2 nm; bandwidth, 1.00 nm; accumulation, 10 times. Liposome solutions with either 1000 μM lipid or PBS were used as blanks. Each experiment was performed in triplicate. Data below 197 nm was not analyzed because of experimental difficulties in obtaining those spectra. Each experiment was performed at least twice.

Artificial membrane calcein permeation measurements. Calcein fluorescence was used to measure peptide-induced liposome permeation. Calcein solutions (10 mM, 20 mM, or 60 mM) containing PC/PG liposomes and peptides were diluted in PBS (×1, ×2, ×3, ×8). PBS (×1, ×2, ×3, ×8) without peptides and PBS (×1, ×2, ×3, ×8) with 10% Triton X-100 were also prepared. Liposome solutions (180 μl) were transferred to wells of black, clear-bottomed 96-well plates (Costar, New York, USA). Peptide solutions (180 μl) and 10% Triton X-100 were transferred to wells of 96-well polyethylene plates (SPL, Gyeonggi-do, Korea). Liposome-containing plates and peptide-containing plates were inserted into a Flexstation 3 Multi-Mode Microplate Reader (Molecular Devices, California, USA). Peptide solutions (20 μl) were transferred and mixed with liposome solution automatically after 30 sec. The mixed solution comprised the following: lipid, 100 μM; peptide, 10 μM, 2 μM, 1 μM, 0.1 μM, or 0 μM. Measurement parameters were as follows: excitation, 490 nm; emission, 520 nm; temperature, 37°C. Triton X-100 (100 μl, 10%) was transferred and mixed with the liposome-peptide solutions automatically after 300 sec to acquire fluorescence at maximum leakage. Fluorescence levels measured 360 sec after addition of the Triton X-100 (1%) control was considered to correspond to 100% permeation. Fluorescence measured between 0–300 sec after addition of 0 μg/ml peptide was considered the baseline. Each experiment was performed at least twice.

Dynamic light scattering (DLS) measurements. Dynamic light scattering was used to measure peptide-induced liposome volume changes. PC/PG-liposome stock solutions and peptides were resuspended in PBS. Liposomes and peptides were mixed to yield these final concentrations: lipid, 100 μM; peptide, either 10 μM or 100 μM. Mixtures were sequentially transferred into a Bladecell disposable cuvette (AvidNano, High Wycombe, England) and measurements were collected with W130i dynamic light scatter (AvidNano, High Wycombe, England) every 10 min, 30 min, 60 min, and 120 min. Measurement parameters were as follows: acquisition, 10; experiment, 10; acquisition time, 180 min; temperature, 20°C. Each sample was measured at least 10 times, and the acquired data were refined by removing outliers (1%). Mean diameter values and polydispersity indices (PDI, the square of the standard deviation / mean diameter) were used to differentiate monodisperse and polydisperse samples. The unpaired t-test was used to compare PDIs of control (PBS-treated) liposomes and sample-treated liposomes.

Supplementary Methods

***In vitro* protease stability**. A mixture of several nonspecific endoproteases and exoproteases (pronase) (Sigma, Darmstadt, Germany) was used to test protease stability. WCopW29, LWCopW29, and colistin were each diluted with PBS (14.0 μg/ml) and mixed with pronase (0.7 μg/ml) in a 50 μl volume. After 0 h, 1 h, 2 h, and 18 h at 37°C, protease activity was stopped by adding trichloroacetic acid, acetonitrile, and urea on ice. The mixtures were then filtered through Millex-GV PVDF-membrane 0.22-μm filters (Merck Millipore Ltd. Darmstadt, Germany). WCopW29 and LWCopW29 samples were individually mixed with protease-stable colistin (as a control). The final mixtures were analyzed by HPLC (Shimadzu, Kyoto, Japan) to compare peak areas (57, 67). To compare relative protease stability, the following equation was used: Peak area (WCopW after x h/colistin after x h) / Peak area (WCopW after 0 h/colistin after 0 h). Each experiment was performed in triplicate.

***In vivo* toxicity tests.** To examine the *in vivo* safety of WCopW43, the peptide was intraperitoneally or subcutaneously injected into 8-week-old female BALB/c mice (n = 3). Peptide doses were 2 mg/mouse (100 mg/kg), 1 mg/mouse (50 mg/kg), or 0.5 mg/mouse (25 mg/kg). The survival rate was monitored for 7 days. All mouse studies were performed following the ethical regulations of the Gwangju Institute of Science and Technology.

A. Matching optimal length with β-strand peptide or α-helix peptide

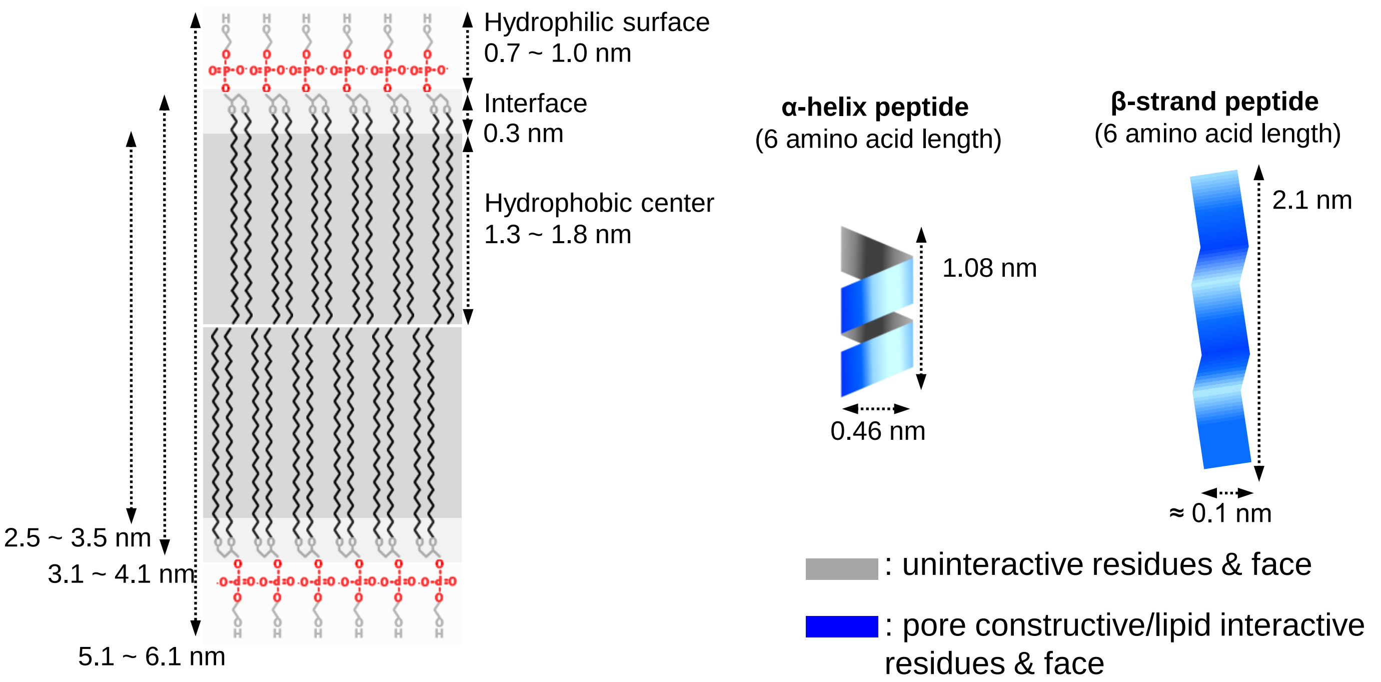

B. Matching gap space with β-strand peptide or α-helix peptide

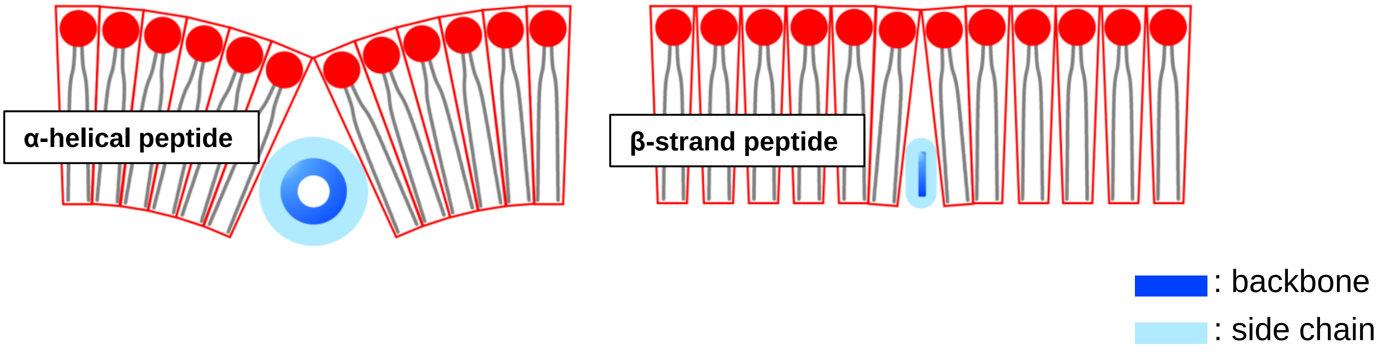

C. Amino acids preferred position in membrane model

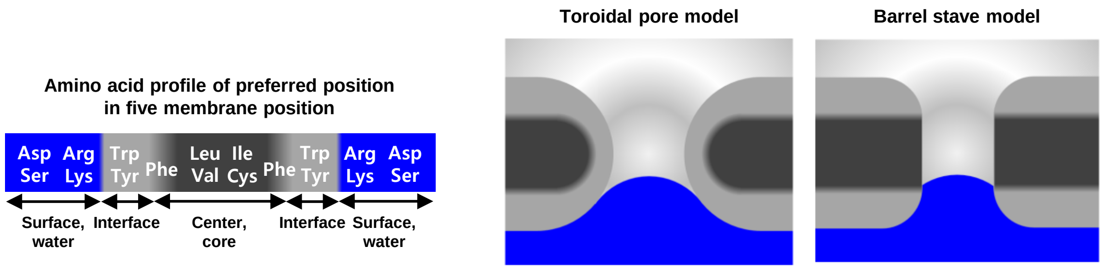

Supplementary Figure 1. AMP–membrane insertion model. The literatures suggested that β-strand AMPs have serial advantages for peptide–membrane interaction because of (a) longer length, (b) thinner diameter. In this paper, we suggest that (c) the preferred position matchable, primary amphipathy is another advantage of β-strand AMPs. Additionally, toroidal pore model allow the placement of Trp3 in the mid-membrane part of a WCopWs.

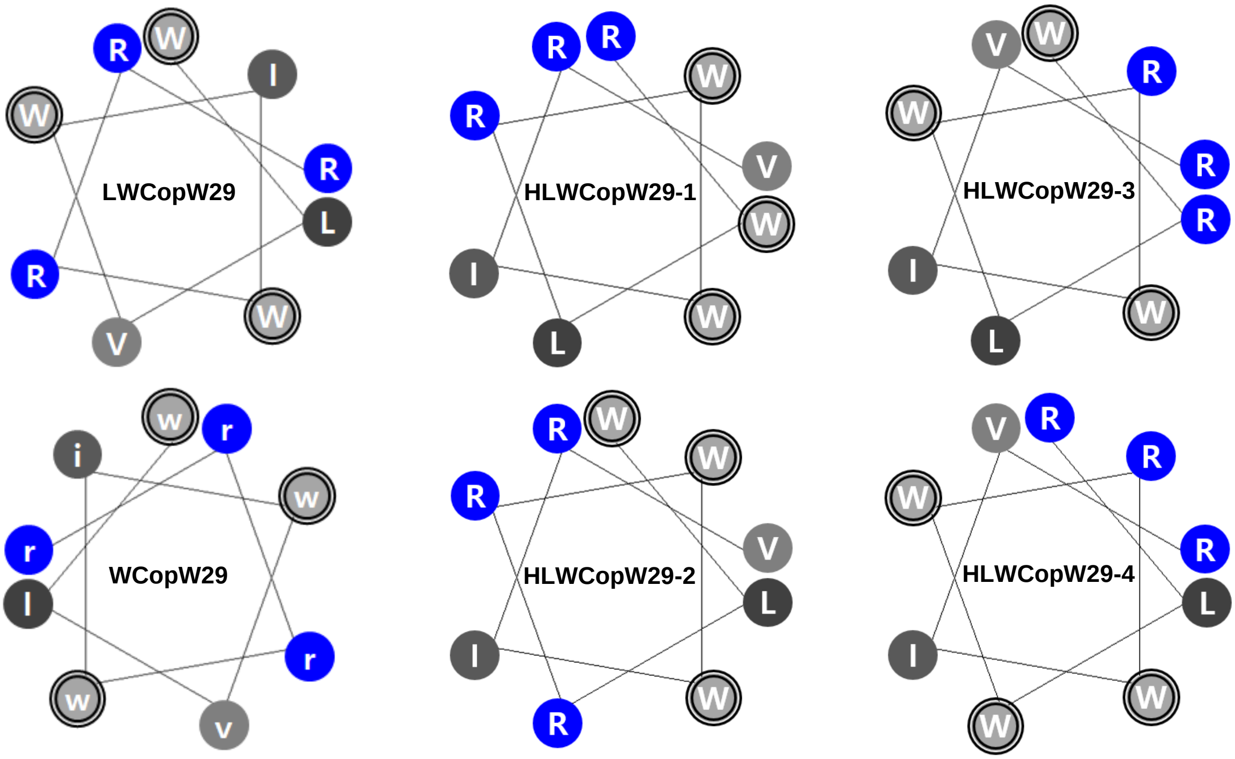

Supplementary Figure 2. Helical wheel diagram of the permutated, racemized LWCopW29 analogs. For easy comparison, circles were marked. The small letters indicate the racemized D-form amino acids, which are assigned on the mirror-reflected helical wheel diagram. The positively charged functional groups are indicated by the blue circles. The aromatic side chains are indicated by the double lined circles. The black and grey circles indicate the carbon branched side chain.

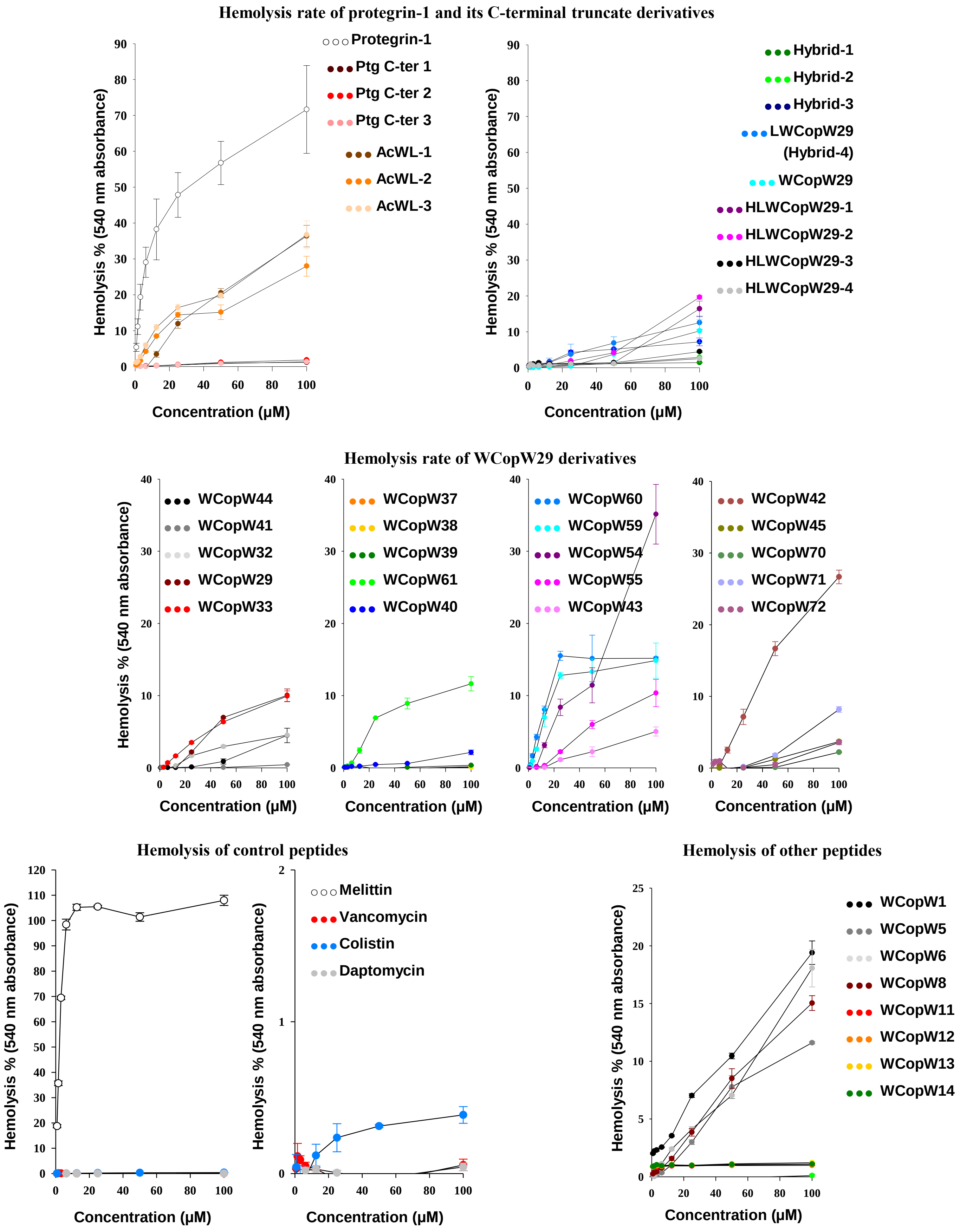

Supplementary Figure 3. Hemolysis activity peptides. The hemolysis rate graph with the standard deviation tested at ten peptide concentrations (100, 50, 25, 12.5, 6.25, 3.13. 1.06 μM) using human red blood cell.

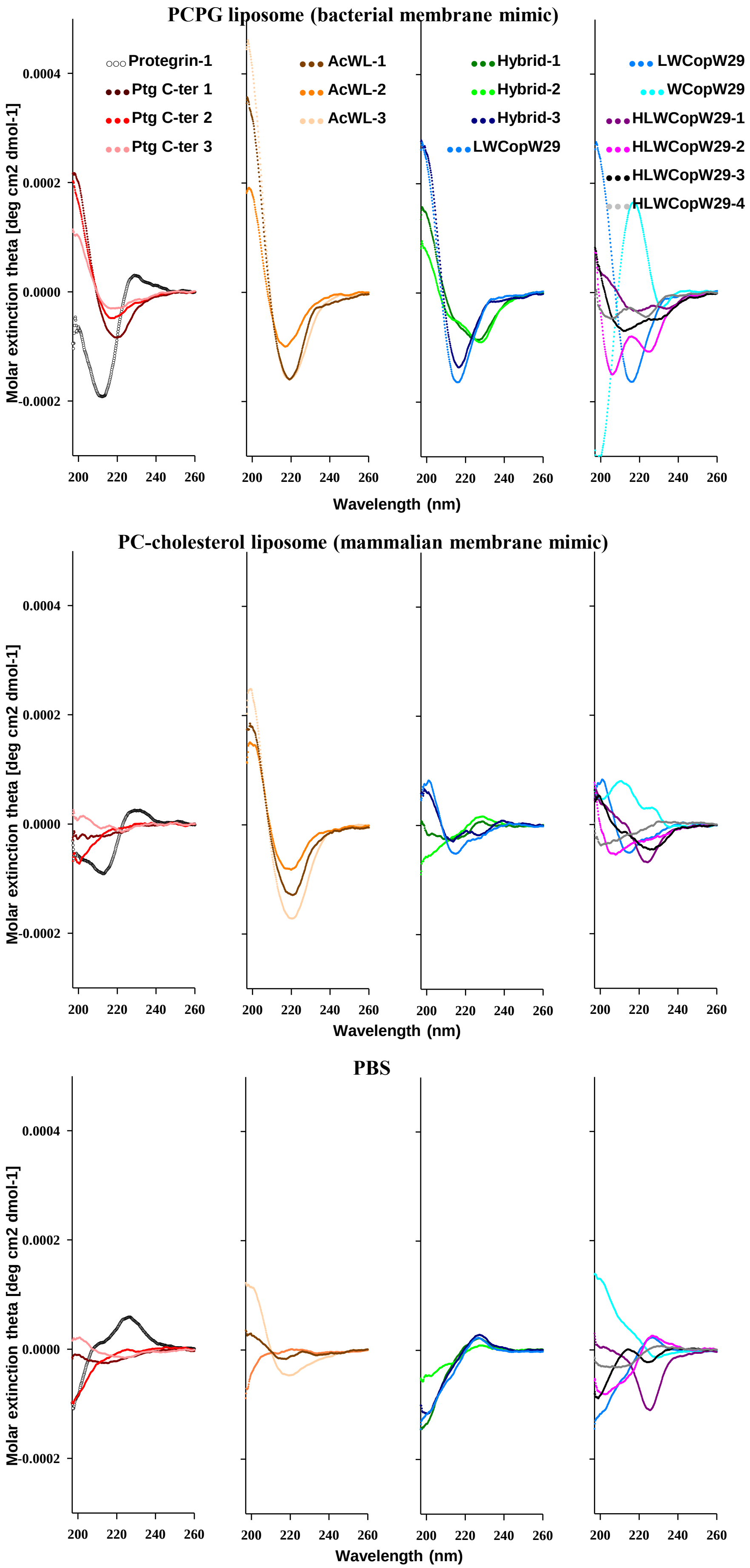
PCPG liposome (bacterial membrane mimic)
Protegrin-1
Ptg C-ter 1
Ptg C-ter 2
Ptg C-ter 3
AcWL-1
AcWL-2
AcWL-3
Hybrid-1
Hybrid-2
Hybrid-3
LWCopW29
LWCopW29
WCopW29
HLWCopW29-1
HLWCopW29-2
HLWCopW29-3
HLWCopW29-4
Molar extinction theta [deg cm2 dmol-1]
0.0004
0.0002
0.0000
-0.0002
200
220
240
260
Wavelength (nm)
PC-cholesterol liposome (mammalian membrane mimic)
PBS

PCPG liposome (bacterial membrane mimic)

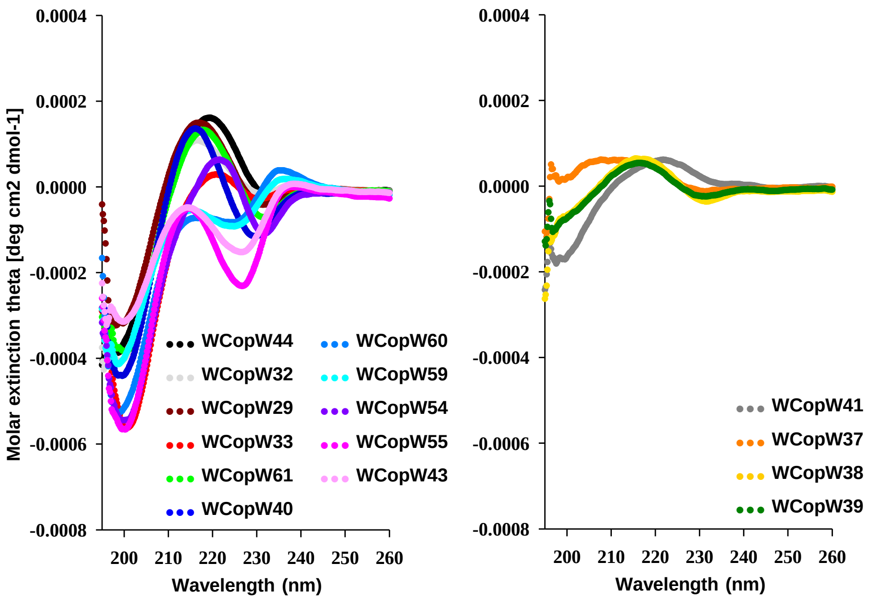

Supplementary Figure 4. Circular dichroism (CD) spectrum. The CD spectrum in the bacterial mimic membrane (DMPC:DMPG liposome), mammalian mimic membrane (DMPC:Cholesterol liposome), and solution (PBS). Negative band around 218 nm indicate β-strand secondary structure. But in the case of the β-sheet AMPs stabilized by disulfide linkages and connected by turns as like the protegrins (1) or arenicin (2), retrocyclin (3), the shift of negative maxima to the 210 nm and the rise of positive maxima around 228 nm observed. Negative bands around 222 nm and 208 nm indicate α-helix secondary structure. Low ellipticity above 210 nm and negative bands around 195 nm indicate disordered peptide. The enantiomeric amino acids (D-forma amino acids) exhibit inversed CD spectrum.

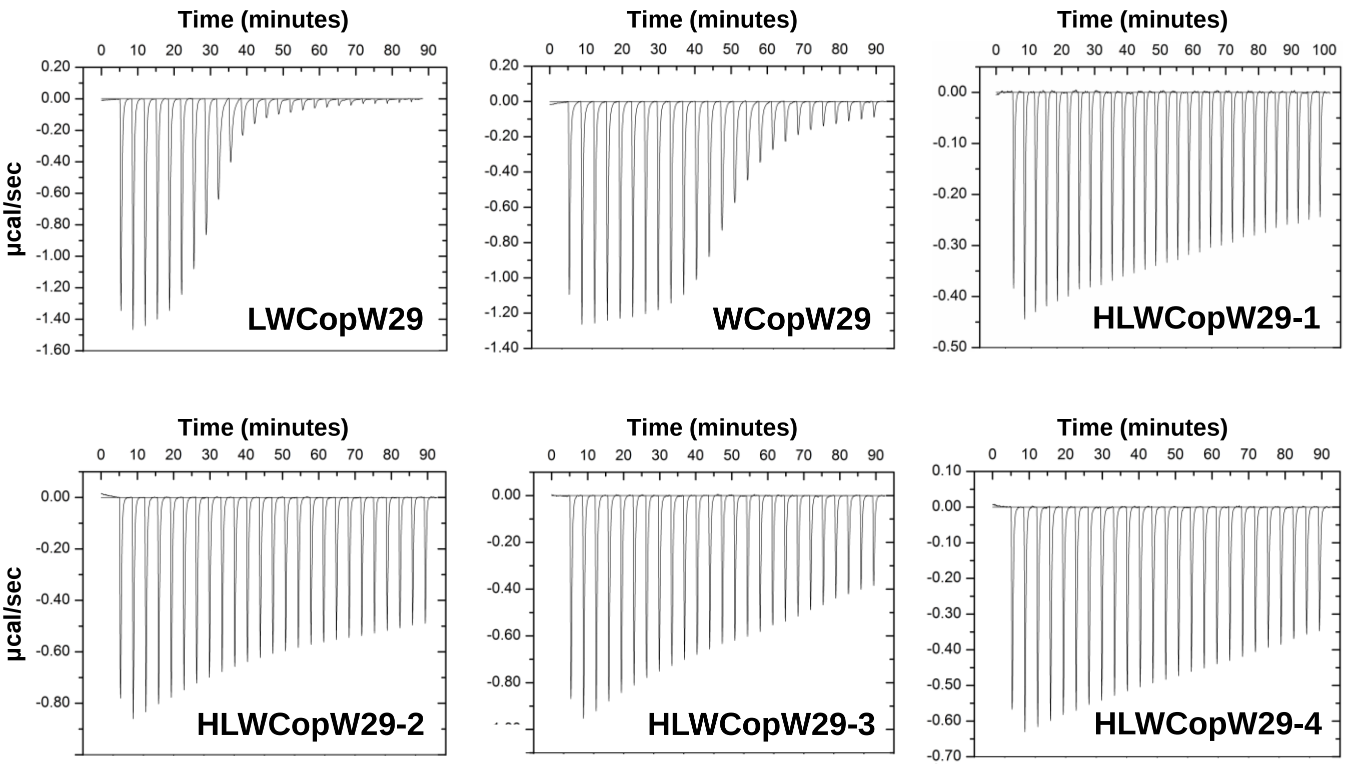

	K (M^{-1})	ΔH (cal/mol)	ΔS (cal/mol deg)
LWCopW29 1st	5.41×10^5	-2.127×10^3	19.4
LWCopW29 2nd	3.94×10^5	-1.910×10^3	19.4
WCopW29	2.19×10^5	-1.934×10^3	18.2
HLWCopW29-1	3.17×10^2	-4.522×10^6	-1.46×10^4
HLWCopW29-2 1st	6.69×10^2	-8.011×10^7	-2.58×10^5
HLWCopW29-2 2nd	6.99×10^2	-3.252×10^7	-1.05×10^5
HLWCopW29-3	1.14×10^3	-3.477×10^7	-1.12×10^5
HLWCopW29-4 1st	4.69×10^2	-1.628×10^7	-5.25×10^4
HLWCopW29-4 2nd	5.90×10^2	-1.125×10^4	-23.6

Supplementary Figure 5. Isothermal titration calorimetry (ITC) thermogram. The isothermal titration calorimetry of the peptide and DMPC:DMPG liposome interaction. ΔH indicate the enthalpy change. ΔS indicate the entropy change. The binding constant, or the association constant K (M-1) is the reverse of the dissociation constant (Kd).

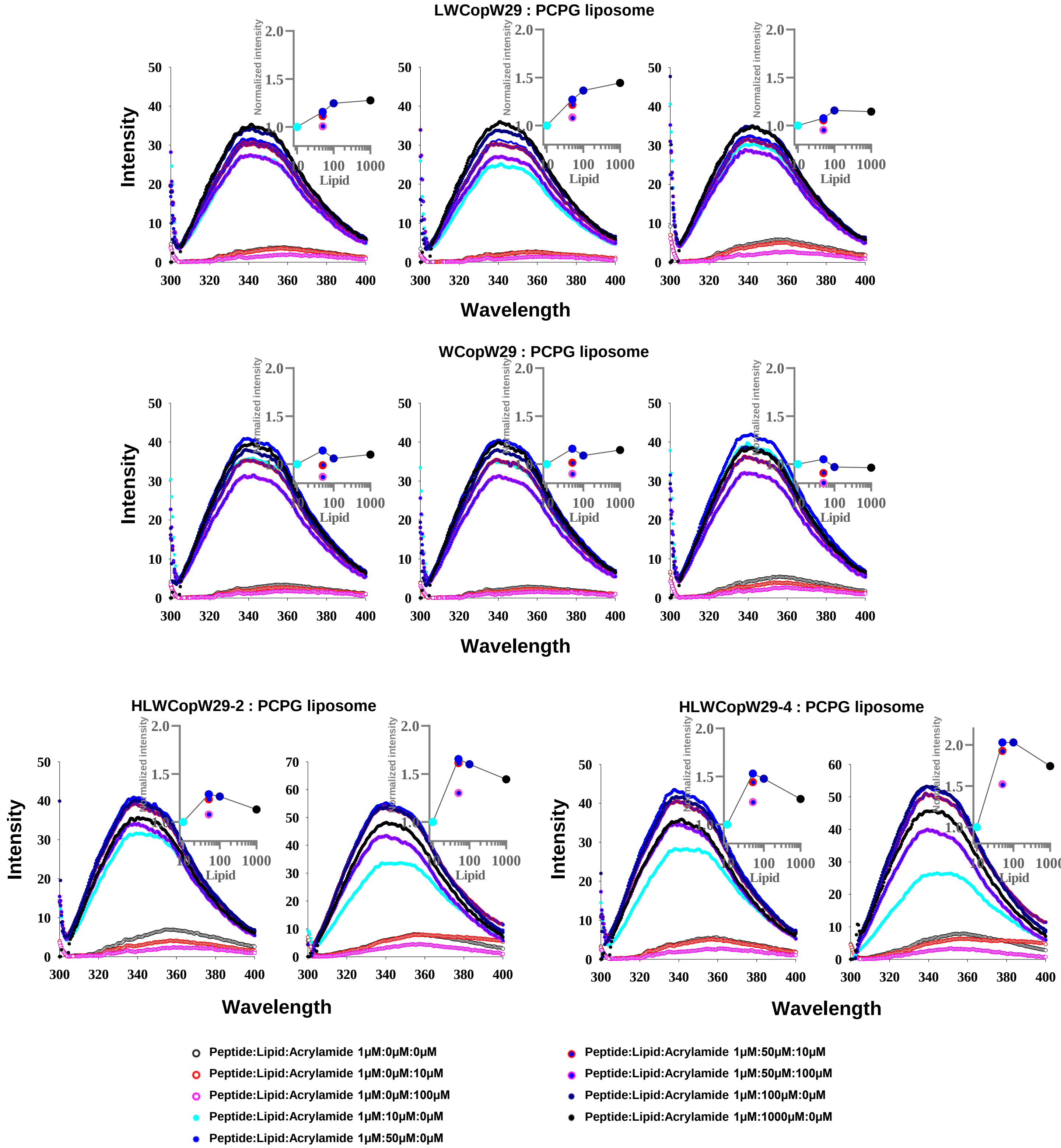

Supplementary Figure 6. Tryptophan fluorescence The graph indicate the tryptophan fluorescence intensity changed by the different DMPC:DMPG liposome and acrylamide with fixed peptide (Trp) concentration. A more intense and blue-shifted fluorescence signal indicates peptide insertion into lipid because the hydrophobic environment of a lipid core prevents fluorescence quenching by water. For easy comparison, The intensity values at the 340 nm maxima are selected and normalized by starting point. Detailed information, such as depth and orientation of insertion, is limited both by tryptophan heterogeneity and by differential fluorescence of D-Trp.

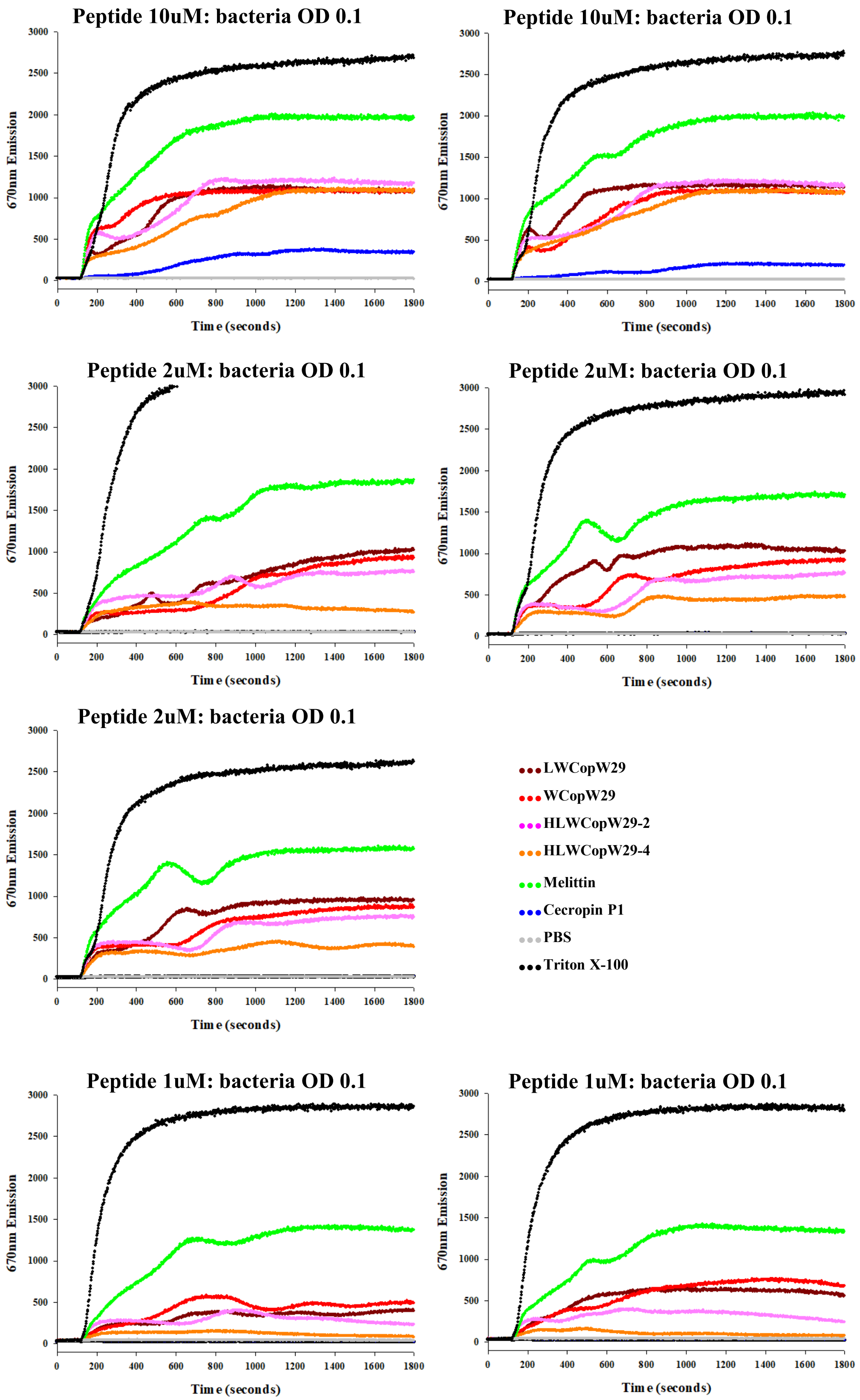
Peptide 10uM: bacteria OD 0.1
Peptide 10uM: bacteria OD 0.1
Peptide 2uM: bacteria OD 0.1
Peptide 2uM: bacteria OD 0.1
Peptide 2uM: bacteria OD 0.1
Peptide 1uM: bacteria OD 0.1
Peptide 1uM: bacteria OD 0.1
670nm Emission
Time (seconds)
0
500
1000
1500
2000
2500
3000
0
200
400
600
800
1000
1200
1400
1600
1800
LWCopW29
WCopW29
HLWCopW29-2
HLWCopW29-4
Melittin
Cecropin P1
PBS
Triton X-100

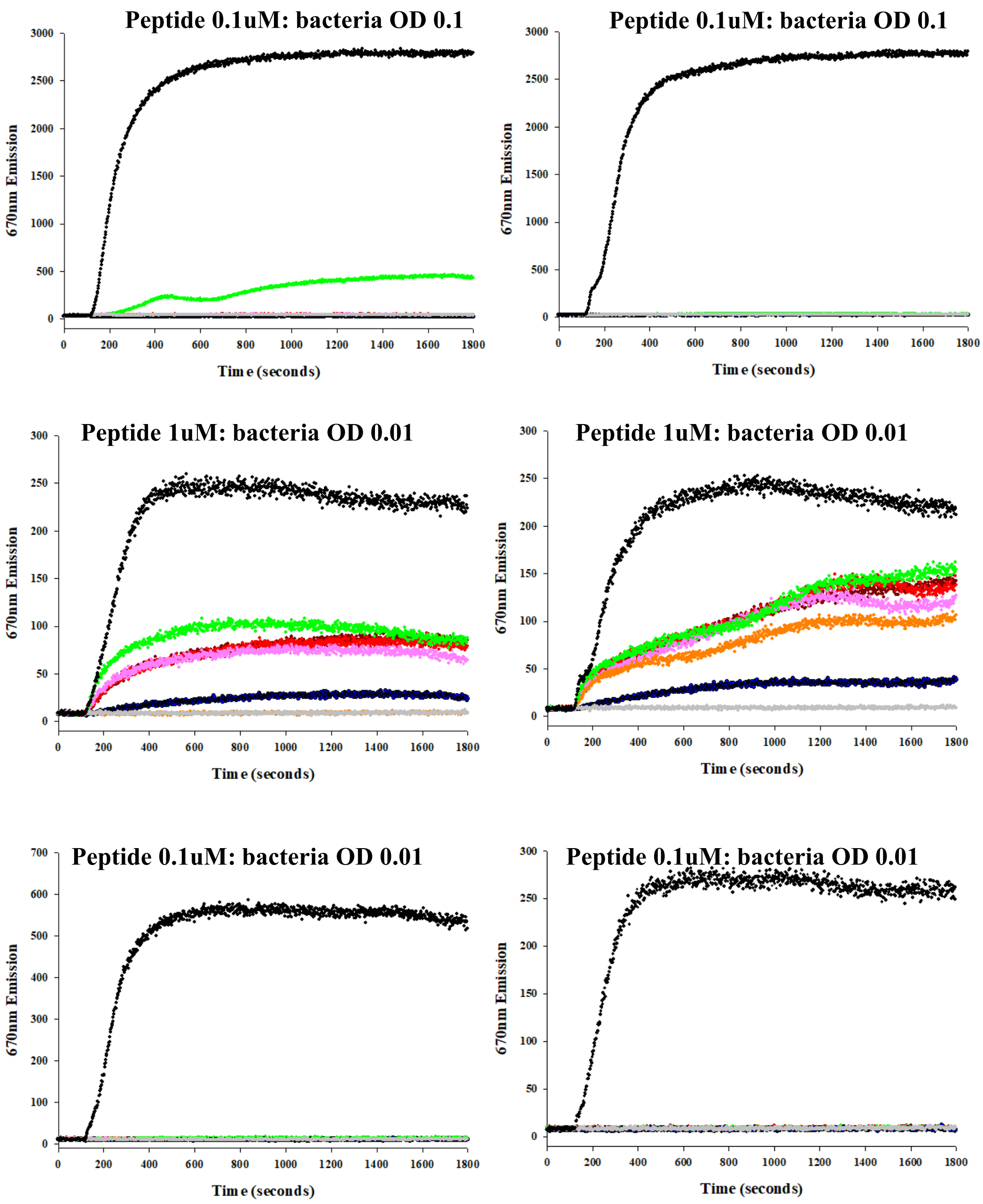

Supplementary Figure 7. Bacteria membrane ion permeation. The H+ leakage induced disc3(5) fluorescence intensity in OD 0.1 or 0.01 *S. aureus*. The MIC of that bacteria concentrations were measured individually [Sup 3]. The time point of peptides addition is 120 sec. The end point is 1800 sec.

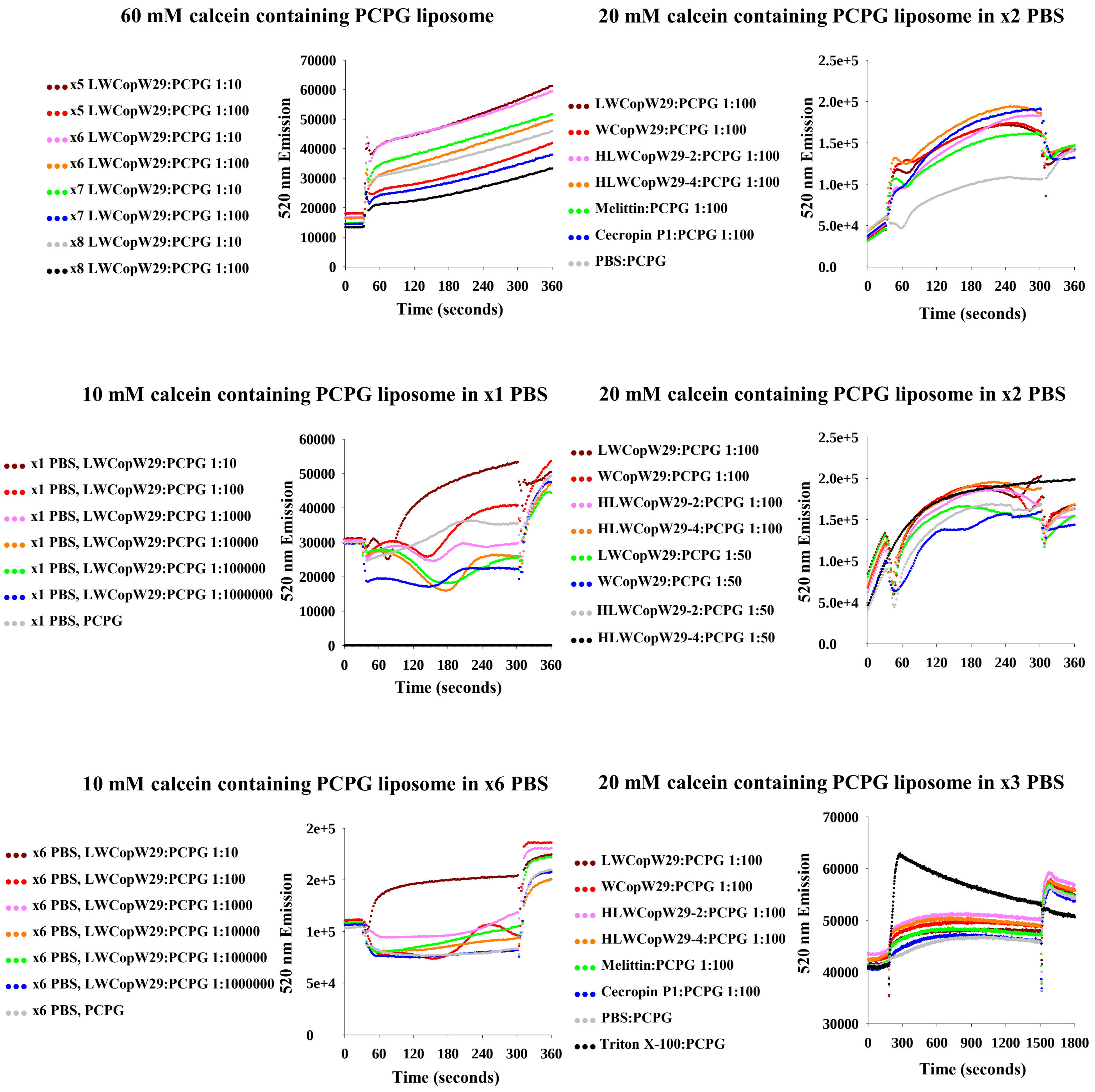

Supplementary Figure 8. Artificial membrane calcein permeation. AMPs induced calcein containing bacterial-mimic liposome permeation test was tried. This test was performed in PBS buffer rather than HEPES buffer. PBS buffer was used to unify all mechanism analysing test experiments in one, fair condition (Hemolysis, CD spectrum, ITC, tryptophan fluorescence, DLS, killing kinetics, OM-IM permeation, SEM imaging). However, calcein containing liposomes are not stabilized in PBS even after serial dilution of the fluorophore and optimization of osmotic-pressure conditions (PBS buffer, 1× to 3×; calcein, 20 mM to ~80 mM). Only high concentration of PBS (6× to 8×), which is too different with other tests, can stabilize calcein containing liposomes. Additionally, calcein lost its quenching property at low concenctration (< 20mM). Therefore, we couldn't analyze AMPs induced calcein containing bacterial-mimic liposome permeation. However, calcein leakage data is shown here because this data could prove the membrane permeation at DLS test condition.

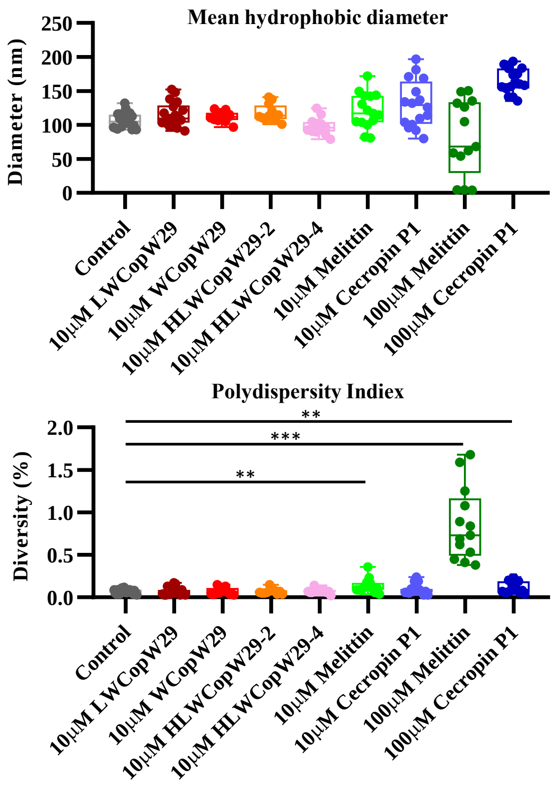

Supplementary Figure 9. Dynamic light scattering (DLS) measured liposome volume change. Changes in liposome volumes (measured by dynamic light scattering) indicated that peptide-induced pore formation made membranes permeable to dye [Fig. S8]. On the one hand, micellization or fusion of liposomes induce dynamic and heterogeneous changes in liposome sizes (a carpet-like mechanism) (4). On the other hand, pore formation in liposomes induces either stable, uniform, and constant-sized liposomes or increases liposome size (5, 6). We considered both mean hydrophobic diameter (MHD) and polydispersity index (PDI) because we could carefully interpret the MHD at high PDI values (> 0.5) (7). All peptide analogs treated liposome volumes are constant MHDs (~100 nm) and PDI values compatible with monodisperse liposomes (< 0.1). Since these values were comparable with values of control liposomes at liposome-leakage-inducing concentrations [Fig. S8], these results indicate that the peptide analogs damaged liposomes by a pore-formation mechanism.

WCopW29 MIC at different cfu/ml

Bacteria concentration	MDR *P. aeruginosa*	MDR *A. baumannii*	MDR *S. aureus*	MDR *E. faecalis*
5 × 10^5 cfu/ml (MIC test condition)	5 μM	0.63 μM	1.25 μM	1.25 μM
2 × 10^8 cfu/ml (x400)	5	2.5	2.5	2.5
1 × 10^{10} cfu/ml (x2000)	No data	10	5	No data

WCopW29 reduced the CFUs of MDR *A. baumannii* and MDR *S. aureus*

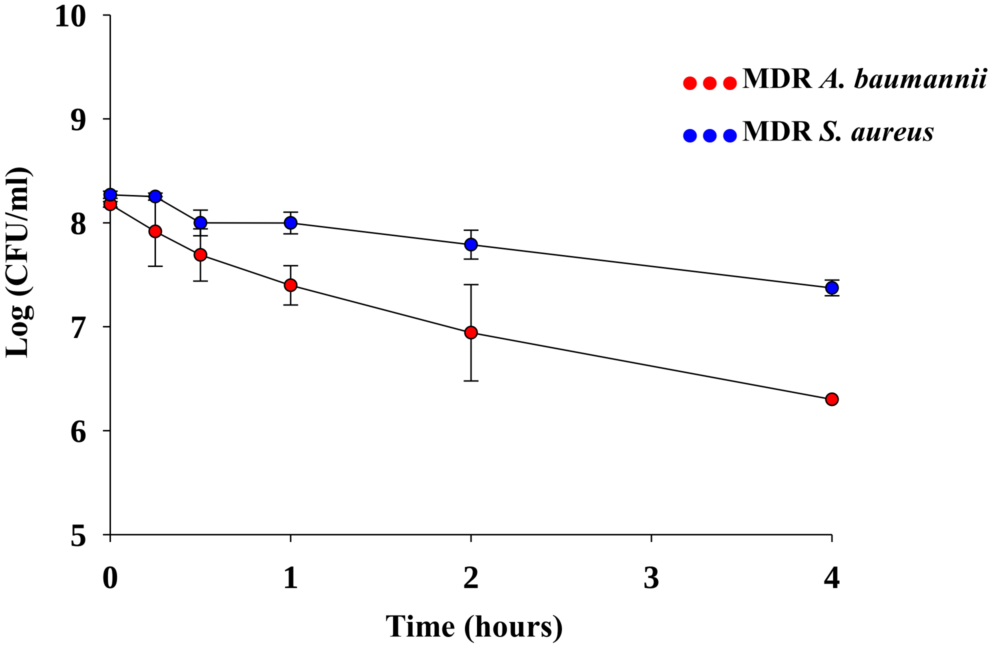

Supplementary Figure 10. Scanning electron microscopy (SEM) test condition. A high concentration of bacteria was required for SEM imaging. Therefore, the MICs at high concentrations of WCopW29 (x400~2000 fold the CLSI MIC test condition) were measured individually. To ensure the reliability of the selected SEM image, killing kinetics in the SEM sample (2 × 10^8 cfu/ml) were measured. After 4 hours of WCopW29 treatment, the loss of colony forming was 90% for MDR *S. aureus* and 99% for MDR *A. baumannii.*

WCopW29 treated MDR *A. baumannii*

x1 MIC 0:00

x1 MIC 0:15

x1 MIC 0:30

x1 MIC 1:00

x1 MIC 4:00

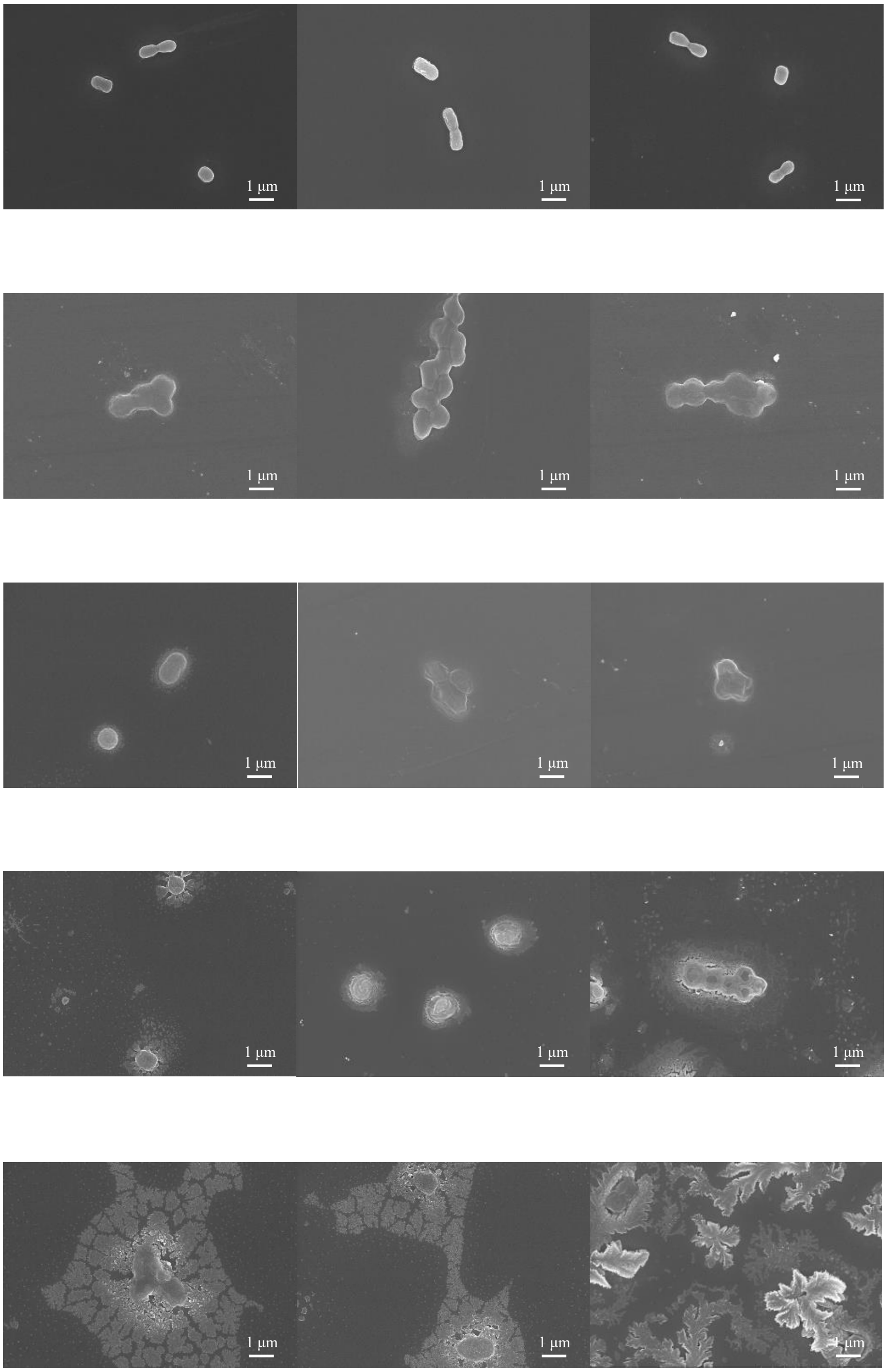

WCopW29 treated MDR *S. aureus*

x1 MIC 0:00

x1 MIC 0:15

x1 MIC 0:30

x1 MIC 1:00

x1 MIC 4:00

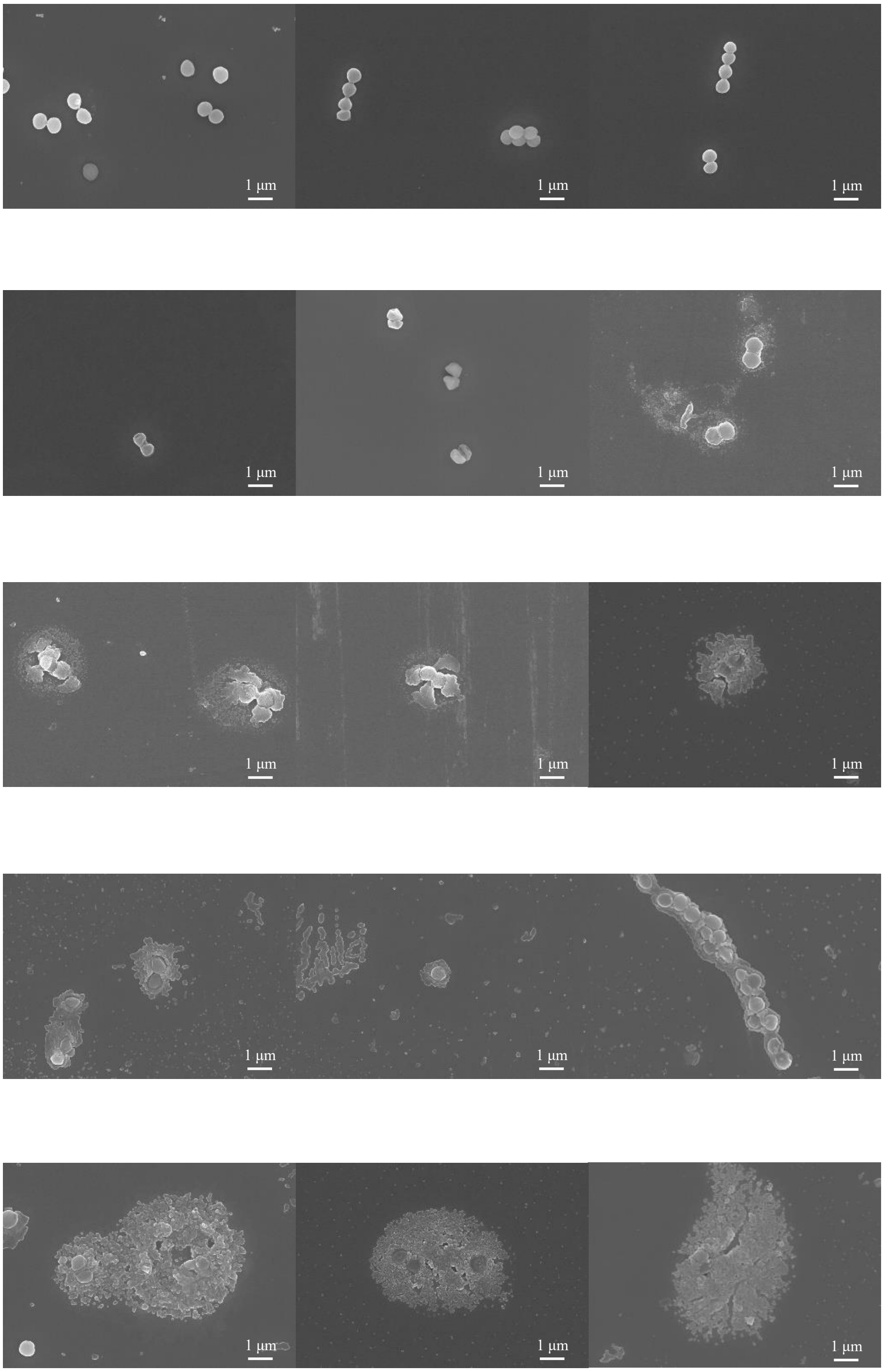

Supplementary Figure 11. Scanning electron microscopy (SEM) image. Time-dependent morphology changes of MDR gram negative and positive bacteria observed by scanning electron microscopy. The morphologies differed depending on the time the image was taken. For example, some morphologies at 0:30 min could be observed at 4-h and vice versa. Under the tested conditions (2 x 10^8 cfu/ml, x1 MIC, PBS), a 4-hour exposure to WCopW29 ensured 90% killing of the bacteria population. Thus, the images taken at 4-h time are representative of the time-dependent changes in morphology.

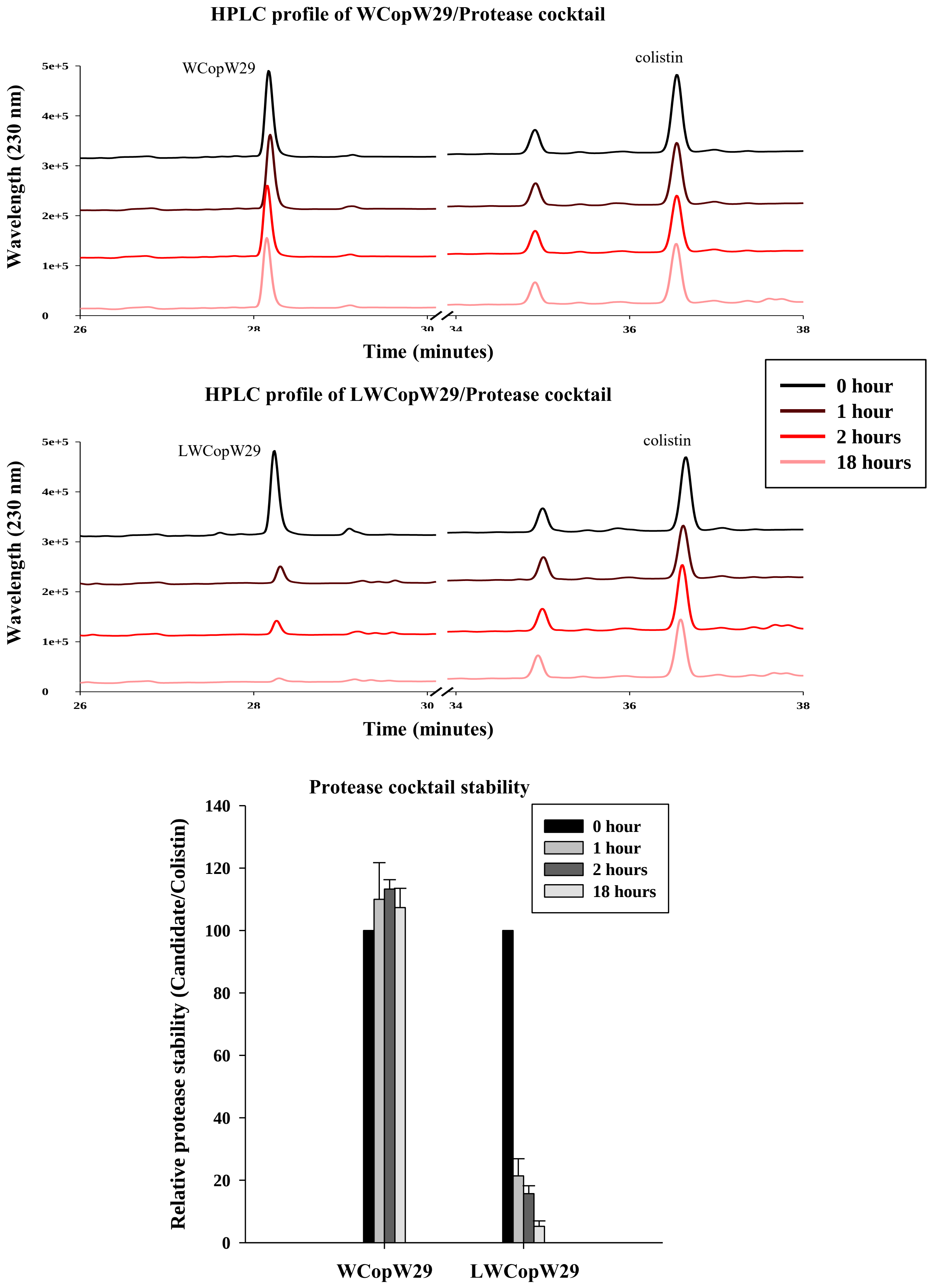

Supplementary Figure 12. Protease stability. Protease cocktail stability was measured by using colistin (protease resistant AMP) as a standard. The following equation was used: 100 × Peak area (WCopW after x hr/colistin after x hr) / Peak area (WCopW after 0 hr/colistin after 0 hr)

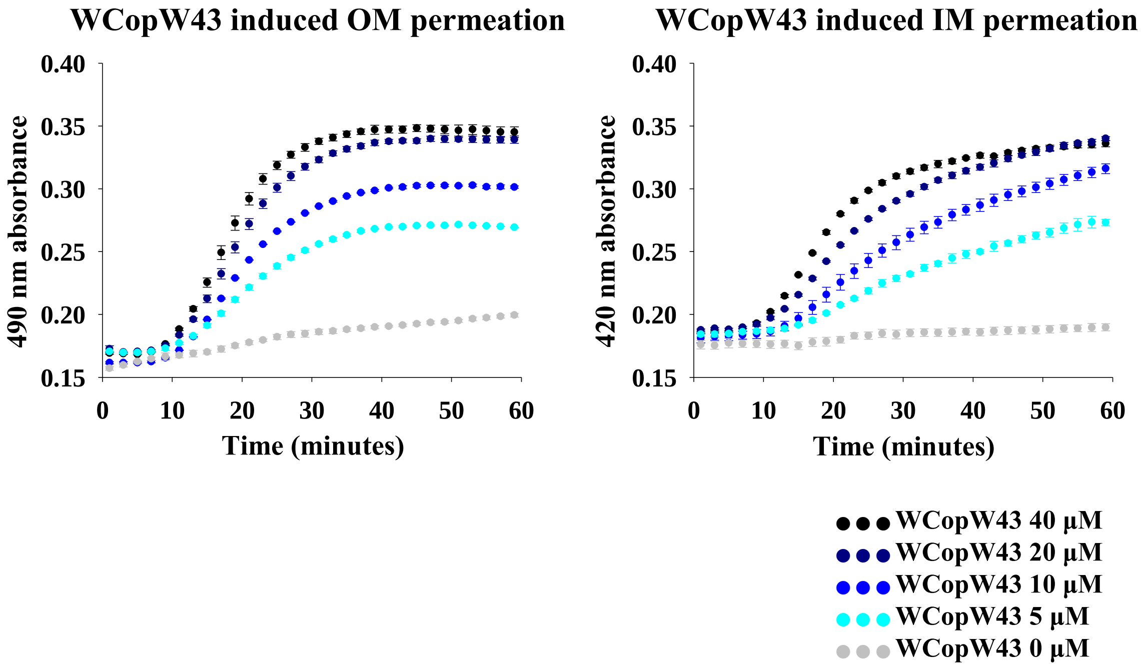

Supplementary Figure 13. OM-IM permeation of WCopW43. The time and concentration dependent outer membrane permeation probed by a nitrocefin absorbance and the inner membrane permeation probed by a ONPG.

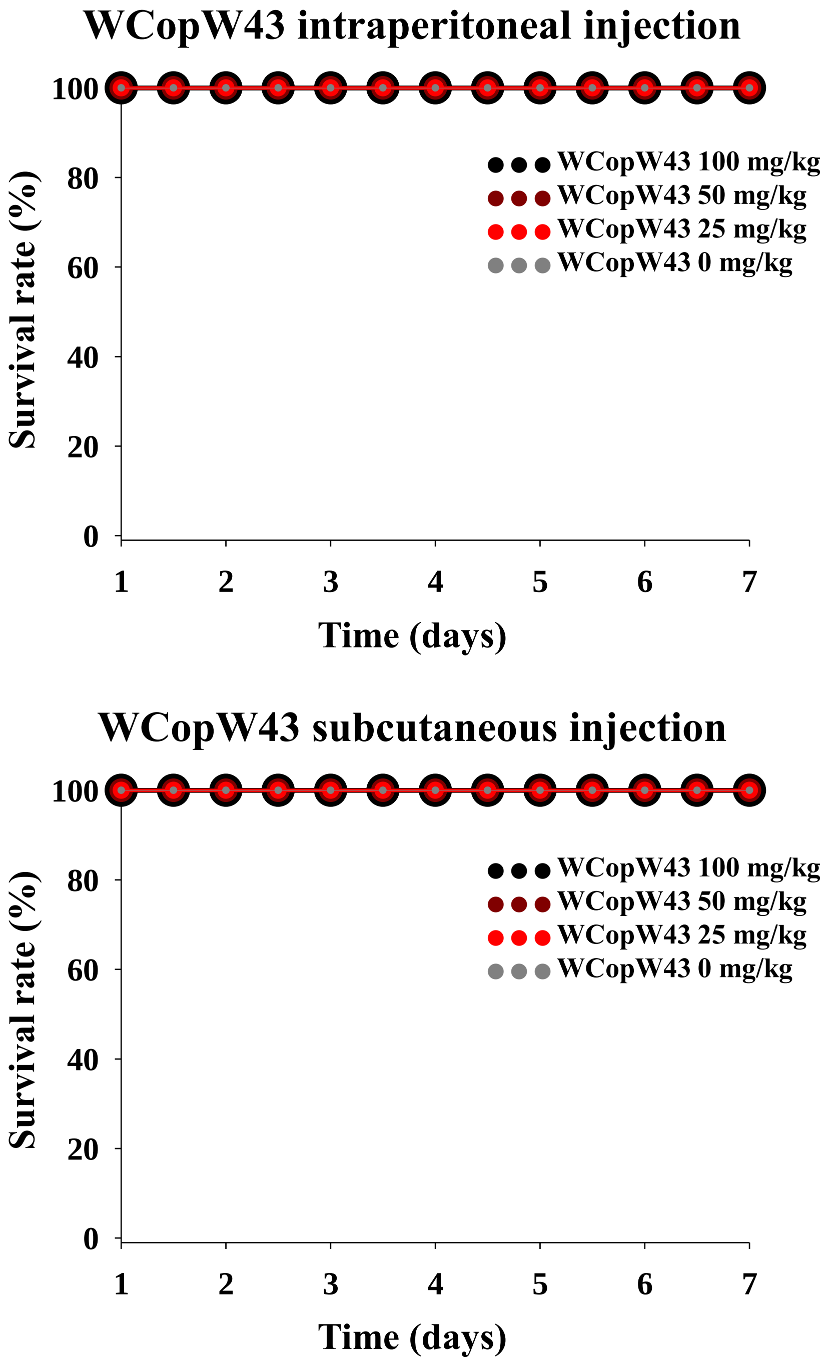

Supplementary Figure 14. *In vivo* toxicity test (survival rate). The survival rate in mouse model. Mouse were intraperitoneally or subcutaneously injected WCopW43 or PBS once. Survival rate was measured every 12 hours for 7 days.

Supplementary Table 1. Sequence, MIC, hemolysis of WCopW analogs.

Compound	Sequence[a]														Minimum inhibitory concentrations (μM)[b]				Hemolysis percent (%)[c]		
															MDR *P. aeruginosa*	MDR *A. baumannii*	MDR *S. aureus*	MDR *E. faecalis*	100 μM	50 μM	25 μM
WtCopW	NH_2		W_t	L	L	W	I	A	L	R	K	K		$CONH_2$	>20	10	5	20		No data	
WhCopW	NH_2		W_h	L	L	W_h	I	A	L	R	K	K		$CONH_2$	>20	>20	5	>20		No data	
WCopW	NH_2		W	L	L	W	I	A	L	R	K	K		$CONH_2$	>20	5	5	10		No data	
WCopW1	NH_2		W	L	L	W	I	A	L	R	K	K	R	$CONH_2$	>20	5	2.5	5	19.40	10.46	7.03
WCopW2	NH_2		W	L	L	W	I	A	L	R	K	*K*	R	$CONH_2$	>20	>20	10	20	23.10	11.43	8.19
WCopW3	NH_2		w	l	l	w	i	a	l	r	k	k	r	$CONH_2$	5	2.5	1.25	2.5	13.37	9.36	3.64
WCopW4	NH_2		w	l	l	w	i	a	l	r	k	*k*	r	$CONH_2$	>20	>20	5	5	18.84	9.85	5.94
WCopW5	NH_2		w	l	l	w	i	g	l	r	k	k	r	$CONH_2$	10	1.25	1.25	2.5	11.60	7.77	2.99
WCopW6	NH_2		w	l	l	w	i	a	l	r	k	k		$CONH_2$	20	2.5	1.25	2.5	18.06	7.05	4.12
WCopW7	NH_2		w	l	l	w	i	a	l	r	k	*k*		$CONH_2$	>20	>20	10	10	14.99	10.05	7.25
WCopW8	NH_2		w	l	l	w	i	g	l	r	k	k		$CONH_2$	>20	5	2.5	5	15.04	8.51	3.85
WCopW9	NH_2	*k*	w	l	l	w	i	g	l	r	k	k	r	$CONH_2$	10	5	1.25	1.25	23.72	19.54	14.30
WCopW10	NH_2	*k*	w	l	l	w	i	g	l	r	k	k	r	$CONH_2$	>20	>20	5	5	23.81	20.39	15.38
WCopW11	NH_2			l	l	w	i	a	l	r	k	k	r	$CONH_2$	20	5	10	5	1.08	1.02	0.94
WCopW12	NH_2			l	l	w	i	a	l	k	k	k	k	$CONH_2$	>20	10	20	20	0.98	0.98	0.98
WCopW13	NH_2			l	l	w	i	g	l	r	k	k	r	$CONH_2$	>20	5	>20	>20	1.21	1.13	1.00
WCopW14	NH_2			l	l	w	i	g	l	k	k	k	k	$CONH_2$	>20	>20	>20	>20	1.10	1.06	0.98
WCopW15	NH_2		w_M	l	l	w	i	g	l	r	k	k	r	$CONH_2$	10	1.25	1.25	2.5	4.04	1.16	0.65
WCopW16	NH_2		w_S	l	l	w	i	g	l	r	k	k	r	$CONH_2$	10	1.25	1.25	2.5	10.59	5.57	2.77
WCopW17								No data								No data				No data	
WCopW18	NH_2		w_F	l	l	w	i	g	l	r	k	k	r	$CONH_2$	5	1.25	1.25	2.5	6.74	4.52	1.89
WCopW19	NH_2	S	w	l	l	w	i	g	l	r	k	k	r	$CONH_2$	10	2.5	2.5	2.5	10.57	7.26	4.32
WCopW20	NH_2		w	a	a	w	i	g	l	r	k	k	r	$CONH_2$	>20	2.5	>20	>20	0.45	0.13	0.11
WCopW21	NH_2		w	l	l	w	a	g	a	r	k	k	r	$CONH_2$	>20	>20	>20	>20	1.74	1.03	0.60
WCopWYL	NH_2		w	l	y	w	i	l	l	r	k	k	r	$CONH_2$	5	1.25	1.25	2.5	9.62	6.29	2.50
WCopWVY	NH_2		w	l	v	w	i	y	l	r	k	k	r	$CONH_2$	5	1.25	1.25	1.25	7.25	4.51	2.47
WCopWVY2	NH_2		w	l	v	w	v	y	l	r	k	k	r	$CONH_2$	10	2.5	2.5	2.5	10.12	6.02	3.46
WCopWVY3	NH_2		w	l	v	w	i	y	v	r	k	k	r	$CONH_2$	10	2.5	2.5	2.5	7.01	5.67	1.92
WCopWVY4	NH_2		w	l	v	w	i	y		r	k	k	r	$CONH_2$	10	1.25	1.25	1.25	5.57	2.57	1.86
WCopW27	NH_2		w	l	i	w	v	y		r	k	k	r	$CONH_2$	5	0.63	1.25	1.25	7.43	3.95	1.82
WCopW28	NH_2		w	l	v	w	i	w		r	r	k	r	$CONH_2$	5	0.63	1.25	1.25	5.26	1.97	2.33
WCopW29	NH_2		w	l	v	w	i	w		r	r	r		$CONH_2$	5	0.63	1.25	1.25	12.00	5.40	0.43
WCopW30	NH_2		w	l	v	w	i	w		q	r	r	r	$CONH_2$	10	0.63	1.25	1.25	4.92	2.74	0.32
WCopW31	NH_2		w	l	v	w	i	w		r	r	q	r	$CONH_2$	10	0.63	1.25	0.63	6.88	1.82	0.35
WCopW32	NH_2		w	l	v	w	i	w		r	r			$CONH_2$	20	2.5	0.63	0.63	4.53	2.95	1.66
WCopW33	NH_2		w	l	v	w	i	y		r	r	r		$CONH_2$	5	0.63	0.63	0.63	9.93	6.35	3.49
WCopW34	NH_2		w	l	v	w	i	w		r	r	e	q	$CONH_2$	>20	20	10	5	-0.14	-0.17	-0.18
WCopW35	NH_2	k	w	l	v	w	i	w		r	r	r		$CONH_2$	2.5	0.63	0.63	0.63	6.07	2.71	1.18
WCopW36	NH_2	d	w	l	v	w	i	w		r	r	r		$CONH_2$	>20	5	1.25	1.25	9.86	7.48	4.03
WCopW37	NH_2		w	l	v	w	k	w		r	r	r		$CONH_2$	>20	2.5	5	5	0.16	0.08	0.13
WCopW38	NH_2		w	l	v	w	d	w		r	r	r		$CONH_2$	>20	>20	>20	>20	0.06	0.06	0.06
WCopW39	NH_2		w	l	v	w	s	w		r	r	r		$CONH_2$	>20	2.5	10	10	0.36	0.06	0.09
WCopW40	NH_2		w	v	v	w	v	w		r	r	r		$CONH_2$	5	0.63	0.63	0.63	2.14	0.59	0.45
WCopW41	NH_2		w	l		w	i	w		r	r	r		$CONH_2$	20	2.5	2.5	1.25	0.42	0.04	0.01
WCopW42	NH_2		w	v	v	w	v	v	w	r	r	r		$CONH_2$	2.5	0.63	0.63	0.63	26.66	16.66	7.14
WCopW43	NH_2		r	r	r	w	i	w	v	l	w	k		$CONH_2$	1.25	0.63	0.63	0.63	5.02	2.22	1.12
WCopW47	NH_2	N	r	r	r	w	i	w	v	l	w	k		$CONH_2$	10	1.25	2.5	2.5	2.07	1.19	0.73
WCopW48	NH_2		r	r	r	w	i	w	v	l	w	k	N	$CONH_2$	2.5	0.63	2.5	2.5	0.85	0.69	0.45
WCopW62	NH_2		R	r	r	w	i	w	v	l	w	k		$CONH_2$	2.5	0.63	1.25	1.25		No data	
WCopW63	NH_2		r	R	r	w	i	w	v	l	w	k		$CONH_2$	2.5	2.5	2.5	2.5		No data	
WCopW64	NH_2		r	r	R	w	i	w	v	l	w	k		$CONH_2$	2.5	2.5	2.5	2.5		No data	
WCopW65	NH_2		R	r	R	w	I	w	V	l	W	k		$CONH_2$	20	>20	20	20		No data	

Compound	Sequence[a]	Minimum inhibitory concentrations (μM)[b]				Hemolysis percent (%)[c]		
		MDR *P. aeruginosa*	MDR *A. baumannii*	MDR *S. aureus*	MDR *E. faecalis*	100 μM	50 μM	25 μM
Melittin		20	1.25	1.25	1.25	97.66	94.83	102.53
Colistin		0.63	0.31	>20	>20	0.39	0.31	0.24
Daptomycin		>20	>20	1.25	1.25	0.04	0.03	0.01

[a] Lower case letters indicate D-form amino acids; capital letters, L-form. The unnatural or modified amino acids indicated by an individual letter; W_t indicate (2,5,7-tri-tert-butyl)tryptophan, W_h indicate 5-Hydroxy-tryptophan, *K,k* of WCopW2,4,7 indicate capric acid attached lysine, *k* of WCopW9 indicate caproic acid attached lysine, *k* of WCopW10 indicate capric acid attached lysine, w_M of WCopW15 indicate methoxy-tryptophan, w_S of WCopW16 indicate benzothienyl-alanine, w_F of WCopW17 indicate fluoro-tryptophan, N of WCopW47, 48 indicate N-acetyl-D-glucosamine attached asparagine.

[b] MIC values were determined in cation-adjusted Mueller-Hinton broth containing 10 mg/l Mg^{2+} and 50mg/l Ca^{2+} under CLSI condition. Multidrug-resistant (MDR) bacterial strains, *P. aeruginosa* CCARM 2180, *A. baumannii* ATCC BAA 1605, *S. aureus* KCCM 40510, and *E. faecalis* ATCC 51575 were used.

[c] Hemolytic activity was tested at three peptide concentrations (100, 50, 25 μM) using 8% human red blood cell.

Supplementary Table 2. MIC (μM) of individual experimental condition

Sample name	Experiment condition (Bacteria concentration)	Experiment condition (Solution)	MIC (*Bacteria,* Strain)			
Colistin	MIC test<br>(5 x 10^5 cfu/ml)	MIC test<br>(CAMHB)	1.25<br>(*P. aeruginosa*, CCARM 2180)	0.63<br>(*A. baumannii*, ATCC BAA 1605)	>20<br>(*S. aureus*, KCCM 40510)	>20<br>(*E. faecalis*, ATCC 51575)
Daptomycin	MIC test<br>(5 x 10^5 cfu/ml)	MIC test<br>(CAMHB)	>5<br>(*P. aeruginosa*, CCARM 2180)	>5<br>(*A. baumannii*, ATCC BAA 1605)	1.25<br>(*S. aureus*, KCCM 40510)	1.25<br>(*E. faecalis*, ATCC 51575)
Vancomycin	MIC test<br>(5 x 10^5 cfu/ml)	MIC test<br>(CAMHB)	>5<br>(*P. aeruginosa*, CCARM 2180)	>5<br>(*A. baumannii*, ATCC BAA 1605)	1.25<br>(*S. aureus*, KCCM 40510)	>20<br>(*E. faecalis*, ATCC 51575)
Melittin	MIC test<br>(5 x 10^5 cfu/ml)	MIC test<br>(CAMHB)	>5<br>(*P. aeruginosa*, CCARM 2180)	1.25<br>(*A. baumannii*, ATCC BAA 1605)	1.25<br>(*S. aureus*, KCCM 40510)	1.25<br>(*E. faecalis*, ATCC 51575)
WCopW29	Killing kinetics<br>(5 x 10^5 cfu/ml)	Killing kinetics<br>(PBS)	5<br>(*P. aeruginosa*, CCARM 2180)	0.63<br>(*A. baumannii*, ATCC BAA 1605)	1.25<br>(*S. aureus*, KCCM 40510)	1.25<br>(*E. faecalis*, ATCC 51575)
LWCopW29<br>(1% DMSO)	TM potential<br>(OD 0.1)	TM potential<br>(CAMHB)			10<br>(*S. aureus*, KCCM 40510)	
WCopW29<br>(1% DMSO)	TM potential<br>(OD 0.1)	TM potential<br>(CAMHB)			2<br>(*S. aureus*, KCCM 40510)	
HLWCopW29-2<br>(1% DMSO)	TM potential<br>(OD 0.1)	TM potential<br>(CAMHB)			>20<br>(*S. aureus*, KCCM 40510)	
HLWCopW29-4<br>(1% DMSO)	TM potential<br>(OD 0.1)	TM potential<br>(CAMHB)			>20<br>(*S. aureus*, KCCM 40510)	
Melittin<br>(1% DMSO)	TM potential<br>(OD 0.1)	TM potential<br>(CAMHB)			20<br>(*S. aureus*, KCCM 40510)	
Cecropin P1<br>(1% DMSO)	TM potential<br>(OD 0.1)	TM potential<br>(CAMHB)			>20<br>(*S. aureus*, KCCM 40510)	
WCopW29	OM-IM permeation<br>(OD 0.2)	OM-IM permeation<br>(CAMHB)	5<br>(E. coli, ML35)			
Melittin	OM-IM permeation<br>(OD 0.2)	OM-IM permeation<br>(CAMHB)	>20<br>(E. coli, ML35)			
WCopW29	SEM sample prepare<br>(1 x 10^8 cfu/ml)	SEM sample prepare<br>(CAMHB)		2.5<br>(*A. baumannii*, ATCC BAA 1605)	2.5<br>(*S. aureus*, KCCM 40510)	
WCopW29	Resistance induction<br>(OD 0.01)	Resistance induction<br>(CAMHB)		1.25<br>(*A. baumannii* KCCM 40203)	1.25<br>(*S. aureus* KCTC 1621)	
Colistin	Resistance induction<br>(OD 0.01)	Resistance induction<br>(CAMHB)		1.25<br>(*A. baumannii* KCCM 40203)	1.25<br>(*S. aureus* KCTC 1621)	
Vancomycin	Resistance induction<br>(OD 0.01)	Resistance induction<br>(CAMHB)		1.25<br>(*A. baumannii* KCCM 40203)	1.25<br>(*S. aureus* KCTC 1621)	
Tetracycline<br>(1% EtOH)	Resistance induction<br>(OD 0.01)	Resistance induction<br>(CAMHB)		1.25<br>(*A. baumannii* KCCM 40203)	1.25<br>(*S. aureus* KCTC 1621)	
Ofloxacin	Resistance induction<br>(OD 0.01)	Resistance induction<br>(CAMHB)		1.25<br>(*A. baumannii* KCCM 40203)	1.25<br>(*S. aureus* KCTC 1621)	
Colistin	MIC test<br>(5 x 10^5 cfu/ml)	MIC test<br>(CAMHB)		0.63<br>(*K. pneumoniae*, NCCP 16125)	>5<br>(*E. aerogenes* NCCP 16285)	
Vancomycin	MIC test<br>(5 x 10^5 cfu/ml)	MIC test<br>(CAMHB)		>5<br>(*K. pneumoniae*, NCCP 16125)	>5<br>(*E. aerogenes* NCCP 16285)	
Melittin	MIC test<br>(5 x 10^5 cfu/ml)	MIC test<br>(CAMHB)		>5<br>(*K. pneumoniae*, NCCP 16125)	>5<br>(*E. aerogenes* NCCP 16285)	
WCopW43	MIC test<br>(5 x 10^5 cfu/ml)	MIC test<br>(CAMHB)		1.25<br>(*K. pneumoniae*, NCCP 16125)	1.25<br>(*E. aerogenes* NCCP 16285)	

MIC measured in the different antibiotics, bacteria, cfu/ml or media, but same incubation condition and times. In case of *S. aureus* KCCM 40510, the bacteria concentration at the TM potential experiment condition (OD 0.1) is 100 fold of MIC condition; 5 x 10^7 cfu/ml.

Supplementary Table 3. MIC in CLSI condition and the modificated CLSI condition for protegrins activity test.

MIC (µM) in CLSI standard condition

Name	MDR *P. aeruginosa*	MDR *A. baumannii*	MDR *S. aureus*	MDR *E. faecalis*
Ptg C-ter 1	>20	>20	>20	>20
Ptg C-ter 2	>20	>20	>20	>20
Ptg C-ter 3	>20	>20	>20	>20
AcWL-1	>20	**2.5**	10	10
AcWL-2	>20	>20	10	10
AcWL-3	>20	**2.5**	5	5
Hybrid-1	>20	>20	>20	>20
Hybrid-2	>20	10	>20	>20
Hybrid-3	>20	**2.5**	10	10
LWCopW29 (Hybrid-4)	>20	**1.25**	5	5
HLWCopW29-1	20	**2.5**	10	10
HLWCopW29-2	>20	5	10	10
HLWCopW29-3	>20	20	20	20
HLWCopW29-4	>20	20	20	20
WCopW29	5	***0.63***	**1.25**	**1.25**
Colistin/Daptomycin	1.25	***0.63***	**1.25**	**1.25**
Protegrin-1	10	10	10	10

MIC (µM) in 0.2% albumin and 0.01% AcOH added CLSI standard condition

Name	MDR *P. aeruginosa*	MDR *A. baumannii*	MDR *S. aureus*	MDR *E. faecalis*
Ptg C-ter 1	>20	>20	>20	>20
Ptg C-ter 2	>20	>20	>20	>20
Ptg C-ter 3	>20	>20	>20	>20
AcWL-1	>20	**1.25**	5	5
AcWL-2	>20	20	10	10
AcWL-3	>20	***0.63***	**2.5**	**2.5**
Hybrid-1	>20	>20	>20	>20
Hybrid-2	>20	10	>20	>20
Hybrid-3	>20	***0.63***	5	2.5
LWCopW29 (Hybrid-4)	20	***0.31***	**1.25**	**1.25**
HLWCopW29-1	20	**1.25**	10	10
HLWCopW29-2	>20	**2.5**	10	10
HLWCopW29-3	>20	20	20	20
HLWCopW29-4	>20	20	20	20
WCopW29	5	***≤0.15***	***0.31***	***0.31***
Colistin/Daptomycin	***≤0.15***	***≤0.15***	***0.31***	***0.31***
Protegrin-1	**1.25**	**1.25**	5	**1.25**

The modified condition containing the 0.2% albumin as a carrier protein and 0.01% acetic acid as a acidifier to reduce the nonspecific binding to plastic. MIC measurement range was modified from 40~0.31 µM to 20~0.15 µM.

Supplementary Table 4. Maximum soluble concentration (μM) in PBS

Name	Protegrin-1	Ptg C-ter 1	Ptg C-ter 2	Ptg C-ter 3	AcWL-1	AcWL-2	AcWL-3	Hybrid-1
Solubility	50	100	100	100	50	50	50	>200

Name	Hybrid-2	Hybrid-3	LWCopW29	WCopW29	HLWCopW29-1	HLWCopW29-2	HLWCopW29-3	HLWCopW29-4
Solubility	>200	>200	>200	>200	>200	>200	>200	>200

Maximum soluble concentrations of peptides in PBS. Protegrin-1 and Ptg C-ter 2,3 are insoluble (< 500 μM) in distilled water.

Supplementary Table 5. Antimicrobial activities of AMPs with the WCOPW29 and LWCOPW29 similar amino-acid compositions (2–3 aromatic-ring side chain amino acids, 3–4 branched-carbon side chain amino acids, and 3–4 cationic side chain amino acids)

Name	Sequence	Net charge	MIC (Strain 1)	MIC (Strain 2)	Experiment condition (Bacteria concentration)	Experiment condition (Solution)	Reference[c]
L3K4W2	NH_2 L K W L K K W L K $CONH_2$	+5	40 µg/ml (*P. aeruginosa* ATCC 27853)	10 µg/ml (*S. aureus* ATCC 6538p)	1×10^{6} cfu/ml	LB	39
L4K3W2	NH_2 L K W L L K W L K $CONH_2$	+4	40 µg/ml (*P. aeruginosa* ATCC 27853)	2.5 µg/ml (*S. aureus* ATCC 6538p)	1×10^{6} cfu/ml	LB	39
Anoplin-1	NH_2 W L L K R W K K L L COOH	+4	8 µM (*P. aeruginosa* ATCC 27853)	32 µM (*S. aureus* ATCC 25923)	5×10^{5} cfu/ml	MHB	40
Anoplin-2	NH_2 w l l k r w k k l l COOH	+4	8 µM (*P. aeruginosa* ATCC 27853)	16 µM (*S. aureus* (ATCC 25923)	5×10^{5} cfu/ml	MHB	40
30	NH_2 K W R R W I R W L $CONH_2$	+5	3.125 µM (*P. aeruginosa* ATCC-CRM9027)	3.125 µM (*S. aureus* ATCC-BAA-1680)	1×10^{6} cfu/ml	CaMHB	41
20	NH_2 R I W V I R W R $CONH_2$	+4	25 µM (*P. aeruginosa* ATCC-CRM9027)	12.5 µM (*S. aureus* ATCC-BAA-1680)	1×10^{6} cfu/ml	CaMHB	41
21	NH_2 R I W V I W R R $CONH_2$	+4	12.5 µM (*P. aeruginosa* ATCC-CRM9027)	6.25 µM (*S. aureus* ATCC-BAA-1680)	1×10^{6} cfu/ml	CaMHB	41
27	NH_2 W K W L K K W I K $CONH_2$	+5	12.5 µM (*P. aeruginosa* ATCC-CRM9027)	12.5 µM (*S. aureus* ATCC-BAA-1680)	1×10^{6} cfu/ml	CaMHB	41
-	NH_2 W K W L K K W I K $CONH_2$	+5	2.9 µM (*P. aeruginosa* H103)	0.7 µM (*S. aureus* ATCC 25923)	1×10^{5} ~ 3.5×10^{5} cfu/ml	MHB	42
-	NH_2 K F K W W R M L I $CONH_2$	+4	2.6 µM (*P. aeruginosa* H103)	1.2 µM (*S. aureus* ATCC 25923)	1×10^{5} ~ 3.5×10^{5} cfu/ml	MHB	42
-	NH_2 R L W W K I W L K $CONH_2$	+4	22.5 µM (*P. aeruginosa* H103)	1.4 µM (*S. aureus* ATCC 25923)	1×10^{5} ~ 3.5×10^{5} cfu/m	MHB	42
-	NH_2 K R R W R I W L V $CONH_2$	+5	3.0 µM (*P. aeruginosa* H103)	1.5 µM (*S. aureus* ATCC 25923)	1×10^{5} ~ 3.5×10^{5} cfu/m	MHB	42
Pac-525	NH_2 K W I K W I K W I $CONH_2$	+4	2 µM (*E. coli* ATCC 25922)	4 µM (*S. aureus* ATCC 29213)	5×10^{5} cfu/ml	MHB	43
Pac-525rev	NH_2 I W R V W R R W K $CONH_2$	+5	4 µM (*E. coli* ATCC 25922)	4 µM (*S. aureus* ATCC 29213)	5×10^{5} cfu/ml	MHB	43
Pac-521	NH_2 K W I K W I K W I $CONH_2$	+4	8 µM (*E. coli* ATCC 25922)	16 µM (*S. aureus* ATCC 29213)	5×10^{5} cfu/ml	MHB	43
Pac-529	NH_2 K W I R W I R W I $CONH_2$	+4	8 µM (*E. coli* ATCC 25922)	4 µM (*S. aureus* ATCC 29213)	5×10^{5} cfu/ml	MHB	43
24,901	NH_2 L R W W W I K R I $CONH_2$	+4	13 µM (*P. aeruginosa* PAO1)	6.3 µM (*S. aureus* ATCC 2592319)	2×10^{5} ~ 7×10^{5} cfu/ml	MHB*	44
74,655	NH_2 A V W K F V K R V $CONH_2$	+4	240 µM (*P. aeruginosa* PAO1)	240 µM (*S. aureus* ATCC 2592319)	2×10^{5} ~ 7×10^{5} cfu/ml	MHB*	44
Tet124	NH_2 K L W W M I R R W $CONH_2$	+4	8 µM (*P. aeruginosa* PAO1)	No data	2×10^{5} ~ 7×10^{5} cfu/ml	MHB	45
Bac8a	NH_2 K I W V I R W R $CONH_2$	+4	16 µM (*P. aeruginosa* PAO1)	16 µM (*S. aureus* ATCC25923	2×10^{5} ~ 7×10^{5} cfu/ml	MHB	46
Bac8b	NH_2 R I W V I R W R $CONH_2$	+4	16 µM (*P. aeruginosa* PAO1)	4 µM (*S. aureus* ATCC25923	2×10^{5} ~ 7×10^{5} cfu/ml	MHB	46
Bac8c	NH_2 R I W V I W R R $CONH_2$	+4	8 µM (*P. aeruginosa* PAO1)	2 µM (*S. aureus* ATCC25923	2×10^{5} ~ 7×10^{5} cfu/ml	MHB	46
Bac8d	NH_2 R R W V I W R R $CONH_2$	+5	250 µM (*P. aeruginosa* PAO1)	16 µM (*S. aureus* ATCC25923	2×10^{5} ~ 7×10^{5} cfu/ml	MHB	46
-	NH_2 I R R R W W W I V $CONH_2$	+4	IC50: 0.23[a] (luciferase expressing *P. aeruginosa*)	No data	Overnight culture of bacteria was diluted 1:500[a]	100 mM Tris buffer, 20 mM glucose[a]	47

* Modified condition containing the 0.2% albumin as a carrier protein and 0.01% acetic acid

[a] Screened antimicrobial activity for the large scale, high-throughput AMPs generation. Each AMPs were synthesized in cellulose support, cleaved, dried and tested directly so exact concentration is uncertain. Antimicrobial activity screened against the genetically engineered luminescent *P. aeruginosa* PAO1. IC50 was validated by a change of luminescence [46, 47]. The IC50 of the most competitive AMPs in this journal (RWRRWKWWL, KRWWKWIRW) are 0.02-0.04 and representative AMPs (RWRIKRWWW, FRRWWKWFK, KIWWWWRKR) are 0.12-0.13 [47].

[c] References in manuscript

Supplementary Table 6. MIC50, MIC90 (μM) against multiple strains

A. baumannii strains	WCopW29	WCopW43	Colistin	Meropenem
Isolated strain 1	**1.25**	**1.25**	***0.63***	>20
Isolated strain 3	**2.5**	**1.25**	>20	>20
Isolated strain 6	**1.25**	***0.63***	**2.5**	>20
Isolated strain 10	**1.25**	***0.63***	**2.5**	>20
Isolated strain 14	**1.25**	**1.25**	**1.25**	>20
Isolated strain 15	**1.25**	***0.63***	***0.63***	***<0.31***
Isolated strain 16	**1.25**	**1.25**	***<0.31***	>20
Isolated strain 19	**1.25**	***0.63***	***<0.31***	>20
Isolated strain 22	**1.25**	***0.63***	**1.25**	>20
Isolated strain 39	**1.25**	***0.63***	***<0.31***	>20
Isolated strain 44	**1.25**	**1.25**	**1.25**	>20
Isolated strain 66	**1.25**	**1.25**	**1.25**	>20
Isolated strain 68	**1.25**	**1.25**	***0.63***	>20
Isolated strain 69	**1.25**	***0.63***	**1.25**	>20
Isolated strain 71	**1.25**	***0.63***	**1.25**	>20
Isolated strain 76	**1.25**	***<0.31***	***<0.31***	>20
Isolated strain 80	***0.63***	***0.63***	***0.63***	>20
Isolated strain 82	**1.25**	***0.63***	***0.63***	>20
Isolated strain 86	***0.63***	***0.63***	***0.63***	>20
Isolated strain 89	**1.25**	**1.25**	**2.5**	>20
Isolated strain 102	**1.25**	***0.63***	***0.63***	>20
Isolated strain 127	**1.25**	**1.25**	***0.63***	>20
Isolated strain 141	**1.25**	***0.63***	***<0.31***	>20
Isolated strain 144	**1.25**	**1.25**	***0.63***	>20
Isolated strain 147	**1.25**	**1.25**	5	>20
Isolated strain 152	**1.25**	***0.63***	**1.25**	>20
KCCM 40203	**1.25**	***0.63***	***0.63***	5
ATCC 17978	**2.5**	**1.25**	***0.63***	***<0.31***
ATCC BBA 1605	***0.63***	**1.25**	***0.63***	>20
NCCP 14654	**1.25**	**1.25**	**1.25**	>20

K. pneumonia strains	WCopW43	Colistin	Meropenem
NCCP 15782	**1.25**	**1.25**	>20
NCCP 15864	**1.25**	***0.63***	>20
NCCP 15866	**2.5**	***0.63***	>20
NCCP 16052	**2.5**	***0.63***	>20
NCCP 16124	**1.25**	20	>20
NCCP 16125	**2.5**	>20	>20
NCCP 16128	5	5	20
NCCP 16208	**1.25**	***<0.31***	>20
NCCP 16212	**2.5**	***<0.31***	>20
NCCP 16213	**1.25**	***0.63***	>20

The isolated strain 1~152 *A. baumannii* strains are isolated and granted from Kyungpook National University School of Medicine. Those strains are commonly resistant to cefotaxime, tobramycin, ciprofloxacin(except isolated strain 22), gentamycin(except isolated strain 22), ceftazidime(except isolated strain 22), tetracycline(except isolated strain 22).

The *A. baumannii* NCCP strains and *K. pneumonia* NCCP strains are purchased from National Culture Collection for Pathogens. NCCP 14654 is resistant to cefepime, ciprofloxacin, gentamicin, aztreonam, levofloxacin, sulfamethoxazole, meropenem. NCCP 15782, 15864, 15866, 16124, 16125 are commonly resistant to aztreonam, amikacin, ciprofloxacin, cefotaxime. All NCCP strains are carbapenem resistant.

Supplementary References

1. Heller WT, Waring AJ, Lehrer RI, Huang HW. Multiple states of beta-sheet peptide protegrin in lipid bilayers. Biochemistry. 37(49):17331-8 (1998)
2. Ovchinnikova TV, Shenkarev ZO, Nadezhdin KD, Balandin SV, Zhmak MN, Kudelina IA, Finkina EI, Kokryakov VN, Arseniev AS. Recombinant expression, synthesis, purification, and solution structure of arenicin. Biochem Biophys Res Commun. 360(1):156-62 (2007)
3. Cole AM, Hong T, Boo LM, Nguyen T, Zhao C, Bristol G, Zack JA, Waring AJ, Yang OO, Lehrer RI. Retrocyclin: a primate peptide that protects cells from infection by T- and M-tropic strains of HIV-1. Proc Natl Acad Sci U S A. 99(4):1813-8104 (2002)
4. Pandidan S, Mechler A. Nano-viscosimetry analysis of the membrane disrupting action of the bee venom peptide melittin. Sci Rep. 9(1):10841 (2019)
5. Wang, Y. Chen, C. H. Hu, D. Ulmschneider, M. B. Ulmschneider, J. P. Spontaneous formation of structurally diverse membrane channel architectures from a single antimicrobial peptide. Nat Commun. 7:13535 (2016)
6. Paterson DJ, Tassieri M, Reboud J, Wilson R, Cooper JM. Lipid topology and electrostatic interactions underpin lytic activity of linear cationic antimicrobial peptides in membranes. Proc Natl Acad Sci USA. 114(40):E8324-E8332 (2017)
7. NanoComposix. Nanocomposix's guide to dynamic light scattering measurement and analysis. V 1.3 (2012)
