# Supplementary material for: Matching amino acids membrane preference profile to improve activity of antimicrobial peptides
Source: Commun Biol. 2022 Nov 8;5:1199. doi: 10.1038/s42003-022-04164-4 (PMC9643456; doi:10.1038/s42003-022-04164-4)
Supplement: Supplementary file 3 — Reporting Summary [file 42003_2022_4164_MOESM3_ESM.pdf]

nature research

Corresponding author(s): Jaeil Kim

Last updated by author(s): Sep 26, 2022

Reporting Summary

Nature Research wishes to improve the reproducibility of the work that we publish. This form provides structure for consistency and transparency in reporting. For further information on Nature Research policies, see our Editorial Policies and the Editorial Policy Checklist.

Statistics

For all statistical analyses, confirm that the following items are present in the figure legend, table legend, main text, or Methods section.

n/a	Confirmed	
☐	☒	The exact sample size (*n*) for each experimental group/condition, given as a discrete number and unit of measurement
☐	☒	A statement on whether measurements were taken from distinct samples or whether the same sample was measured repeatedly
☐	☒	The statistical test(s) used AND whether they are one- or two-sided<br>*Only common tests should be described solely by name; describe more complex techniques in the Methods section.*
☐	☒	A description of all covariates tested
☐	☒	A description of any assumptions or corrections, such as tests of normality and adjustment for multiple comparisons
☐	☒	A full description of the statistical parameters including central tendency (e.g. means) or other basic estimates (e.g. regression coefficient) AND variation (e.g. standard deviation) or associated estimates of uncertainty (e.g. confidence intervals)
☐	☒	For null hypothesis testing, the test statistic (e.g. *F*, *t*, *r*) with confidence intervals, effect sizes, degrees of freedom and *P* value noted<br>*Give P values as exact values whenever suitable.*
☒	☐	For Bayesian analysis, information on the choice of priors and Markov chain Monte Carlo settings
☒	☐	For hierarchical and complex designs, identification of the appropriate level for tests and full reporting of outcomes
☒	☐	Estimates of effect sizes (e.g. Cohen's *d*, Pearson's *r*), indicating how they were calculated

Our web collection on statistics for biologists contains articles on many of the points above.

Software and code

Policy information about availability of computer code

Data collection	SoftMax Pro Software, MicroCal PEAQ-ITC family Analysis software and FL WinLab™ software were used for data collection
Data analysis	Graphpad prism software, Sigmaplot software and Microsoft Excel software were used for collected data analysis

For manuscripts utilizing custom algorithms or software that are central to the research but not yet described in published literature, software must be made available to editors and reviewers. We strongly encourage code deposition in a community repository (e.g. GitHub). See the Nature Research guidelines for submitting code & software for further information.

Data

Policy information about availability of data

All manuscripts must include a data availability statement. This statement should provide the following information, where applicable:

- Accession codes, unique identifiers, or web links for publicly available datasets
- A list of figures that have associated raw data
- A description of any restrictions on data availability

Provide your data availability statement here.

Field-specific reporting

Please select the one below that is the best fit for your research. If you are not sure, read the appropriate sections before making your selection.

☒ Life sciences ☐ Behavioural & social sciences ☐ Ecological, evolutionary & environmental sciences

For a reference copy of the document with all sections, see nature.com/documents/nr-reporting-summary-flat.pdf

Life sciences study design

All studies must disclose on these points even when the disclosure is negative.

Sample size	For brief in vitro antibiotics antimicrobial activity screening, 4 bacteria strains was used as sample. For detailed in vitro activity analysis for promising antibiotics candidates, 45 bacteria strains was used as sample. For in vivo activity test, 10 mice were used to test antibiotics activity against each bacteria strain.
Data exclusions	No data were excluded from the analysis
Replication	All in vitro activity studies were repeated at least 3 times. All in vitro mode of action studies were repeated 2-3 times. All attempts of replication were reproducible.
Randomization	We purchased and used the precise number of mice for in vivo experiments as IACUC (Institutional Animal Care and Use Committee) guideline. Therefore we couldn't randomize animals for in vivo experiments.
Blinding	Blinding is not used because we cannot results of experiment before acquiring data

Reporting for specific materials, systems and methods

We require information from authors about some types of materials, experimental systems and methods used in many studies. Here, indicate whether each material, system or method listed is relevant to your study. If you are not sure if a list item applies to your research, read the appropriate section before selecting a response.

Materials & experimental systems

n/a	Involved in the study	
☒	☐	Antibodies
☒	☐	Eukaryotic cell lines
☒	☐	Palaeontology and archaeology
☐	☒	Animals and other organisms
☒	☐	Human research participants
☒	☐	Clinical data
☐	☒	Dual use research of concern

Methods

n/a	Involved in the study	
☒	☐	ChIP-seq
☒	☐	Flow cytometry
☒	☐	MRI-based neuroimaging

Animals and other organisms

Policy information about studies involving animals; ARRIVE guidelines recommended for reporting animal research

Laboratory animals	Female BALB/c mice (8-week old) were used
Wild animals	No wild animals used
Field-collected samples	Field-collected samples used
Ethics oversight	All experiments oversighted by the IACUC (Institutional Animal Care and Use Committee) guideline of each institution.

Note that full information on the approval of the study protocol must also be provided in the manuscript.

Dual use research of concern

Policy information about dual use research of concern

Hazards

Could the accidental, deliberate or reckless misuse of agents or technologies generated in the work, or the application of information presented in the manuscript, pose a threat to:

No	Yes	
☐	☒	Public health
☒	☐	National security
☒	☐	Crops and/or livestock
☒	☐	Ecosystems
☒	☐	Any other significant area

Hazards: This experiments used multi-drug resistant bacteria.

For examples of agents subject to oversight, see the United States Government Policy for Institutional Oversight of Life Sciences Dual Use Research of Concern.

Experiments of concern

Does the work involve any of these experiments of concern:

No	Yes	
☒	☐	Demonstrate how to render a vaccine ineffective
☐	☒	Confer resistance to therapeutically useful antibiotics or antiviral agents
☐	☒	Enhance the virulence of a pathogen or render a nonpathogen virulent
☒	☐	Increase transmissibility of a pathogen
☒	☐	Alter the host range of a pathogen
☒	☐	Enable evasion of diagnostic/detection modalities
☒	☐	Enable the weaponization of a biological agent or toxin
☒	☐	Any other potentially harmful combination of experiments and agents

Precautions and benefits

Biosecurity precautions	Multi-drug resistant bacteria were handled with protective equipment in biosafety level II laboratory. Hazardous wastes were autoclaved.
Biosecurity oversight	All experiments oversighted by the Safety management team of each institution.
Benefits	This experiments developed strong antibiotics candidates against multi-drug resistant bacteria.
Communication benefits	This experiments developed strong antibiotics candidates against multi-drug resistant bacteria.
